# Association of adverse childhood experiences and gastrointestinal and liver diseases among middle-aged and elderly adults in China

**DOI:** 10.1017/S2045796026100729

**Published:** 2026-07-20

**Authors:** Yuanfeng Zhang, Chenglong Li, Zilun Wu, Yang Cao, Zumin Shi

**Affiliations:** 1 First Affiliated Hospital of Guangzhou University of Chinese Medicine, Guangzhou University of Chinese Medicine, Guangzhou, China; 2 Postdoctoral Research Center, Guangzhou University of Chinese Medicine, Guangzhou, China; 3 National Institute of Health Data Science at Peking University, Beijing, China; 4 Human Nutrition Department, College of Health Sciences, QU Health, Qatar University, Doha, Qatar

**Keywords:** adverse childhood experiences (ACEs), cohort study, depressive symptoms, epidemiology, gastrointestinal diseases, liver diseases, loneliness, mediation

## Abstract

**Aims:**

Research on the link between threat-related and deprivation-related adverse childhood experiences (ACEs) and the risk of gastrointestinal (GI) and liver diseases in later life remains limited. This study aims to evaluate the independent associations of threat-related and deprivation-related ACEs with the development of GI and liver disorders in middle-aged and older Chinese adults.

**Methods:**

This prospective cohort study used data from the China Health and Retirement Longitudinal Study, which included participants aged 45 and older who had complete ACE data, two health assessments, and no pre-existing GI or liver conditions at baseline. Participants reported on five threat-related and deprivation-related ACEs before age 17. GI and liver diseases were classified based on self-reported physician diagnoses.

**Results:**

The outcomes of GI and liver diseases are based on self-reported physician diagnosis and broad categories. Compared with no exposures, participants with two or more threat-related ACEs exhibited a higher risk of both chronic liver disease (hazard ratio [HR], 1.26; 95% confidence interval [CI], 1.04–1.52; *P* = 0.016) and GI disease (HR, 1.36; 95% CI, 1.20–1.55; *P* < 0.001); two or more deprivation-related ACEs showed stronger associations with GI disease (HR, 1.48; 95% CI, 1.28–1.70; *P* < 0.001); and no significant associations with liver disease risk across all exposure levels. Additionally, depressive symptoms accounted for 10.7% (*P* = 0.003) of the association between threat-related ACEs and liver disease risk and accounted for 12.7% (*P* < 0.001) of the association between threat-related ACEs and GI disease risk. Midlife loneliness accounted for 5.3% (*P* = 0.001) of the association between threat-related ACEs and incident GI diseases and for 3.7% (*P* = 0.004) of the association between deprivation-related ACEs and incident GI diseases.

**Conclusions:**

Threat-related ACEs are directly associated with an increased risk of liver and GI diseases. A modest proportion of this observed relationship is partially mediated through depressive symptoms and loneliness in middle age.

## Introduction

Gastrointestinal (GI) and liver diseases share a common pathogenic basis rooted in dysregulated immune-inflammatory responses (Schwärzler *et al.*, 2026), justifying their combined analysis in this study and hinting at potential overlapping aetiologies. Together, digestive diseases (including GI and chronic liver diseases) represent a massive global health burden, responsible for over 2.8 billion prevalent cases and 8 million deaths annually worldwide. This burden disproportionately falls on low- and middle-income countries (Wang *et al.*, 2023), highlighting the need for focused research in major nations like China. The prevalence of GI disease in the Chinese population is between 2.3% and 15.8% (Liu and Liu, 2021). Liver disease burden is heavy and rising, and the prevalence increased in China from 23.8% to 29% (Devarbhavi *et al.*, 2023). Identifying risk factors for GI and liver diseases is crucial to designing public health interventions and promoting healthy aging in China.

Recent studies indicate that adverse childhood experiences (ACEs) significantly contribute to GI or liver disorders throughout life (Siewert-markus *et al.*, 2024; Lee *et al.*, 2025). ACEs refer to a wide range of intensive stressors that occur before the age of 18, such as emotional neglect and physical abuse. These early-life adversities affect neurodevelopment, impairing social, emotional and psychological functioning and increasing long-term health risks (Hughes *et al.*, 2021). However, ACEs are divided into threat and deprivation because they tap into fundamentally different neurobiological pathways. Use of the total ACE score obscures the unique influence of different childhood adversity types on mental health and cognitive development, threat via stress-system hyper-activation and fear-circuitry calibration, and deprivation via the disruption of experience-dependent synaptic pruning and cortical development (Mitchell *et al.*, 2025). However, most prior studies in this field adopt a cross-sectional design, which precludes establishing temporal causality and assessing incident outcomes, and the differential associations of these two ACE dimensions with incident GI and liver diseases in middle-aged and older Chinese adults remain poorly elucidated.

Increasing research has focused on identifying modifiable downstream pathways that may mitigate the risks associated with historically fixed ACE exposures (Sonu *et al.*, 2019; Li *et al.*, 2022c). Evidence suggests that psychological factors are associated with liver and GI diseases (Warreman *et al.*, 2023; Lee *et al.*, 2025). Nevertheless, the mediating role of depressive symptoms and loneliness in the associations between domain-specific ACEs and incident GI/liver diseases has not been well-characterized.

This cohort study used data from the China Health and Retirement Longitudinal Study (CHARLS) to explore how ACEs related to threat and deprivation are linked to GI and hepatic disorders in middle-aged and older Chinese adults. Additionally, we evaluated whether depressive symptoms and loneliness mediate these associations, elucidating mechanistic pathways between early adversity and adult diseases.

## Methods

### Study design and population

This cohort study utilized data from the CHARLS baseline survey (2011), with follow-up through waves 1–4 (2011–2018). CHARLS is a nationally representative longitudinal study examining aging-related health and socioeconomic factors among middle-aged and older Chinese adults. The study received ethical approval from the Biomedical Ethics Review Committee of Peking University (IRB00001052-11015), and all participants provided written informed consent. The research followed the STROBE guidelines for reporting observational studies.

**Figure 1.**
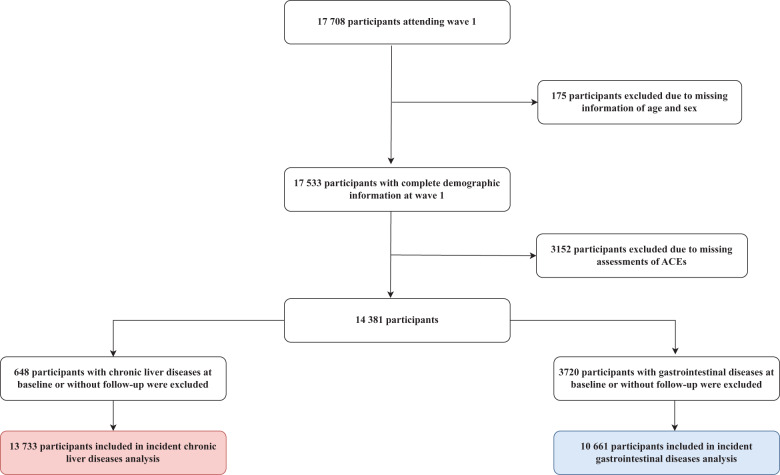
Participants’ selection diagram. Figure 1 long description.

The study initially enrolled 17,708 participants from 450 villages/resident communities across 28 Chinese provinces using a multistage probability sampling strategy. After excluding 175 participants with missing age/sex data and 3,152 with incomplete ACEs assessments, 14,381 remained. Further exclusions included 648 participants with prevalent chronic liver diseases and 3,720 with GI diseases at baseline or lost to follow-up. The final sample included 13,733 participants for incident chronic liver disease analysis and 10,661 for incident GI disease analysis. Participant selection details are shown in Fig. 1. This methodological approach is consistent with contemporary large-scale cohort studies, which utilized independent datasets for multiple outcomes to ensure statistical power (Wei *et al.*, 2026).

### GI and liver disease measurements

The outcome variables are based on broad, self-reported categories. The diagnosis of GI and liver diseases was ascertained using self-reported data from baseline and follow-up health questionnaires. Participants were specifically asked: ‘Has a doctor ever diagnosed you with a digestive disease (excluding tumours or cancers)?’ and ‘Has a doctor ever diagnosed you with a liver disease (excluding fatty liver, cancers or tumours)?’ Affirmative responses to either question classified the respondent as having a GI or liver disease. For participants with new-onset GI or liver diseases, years to disease onset were calculated from the age of attending baseline survey until the age of first incident disease diagnosis, while those without GI and liver diseases were censored, with censoring time calculated from the age of attending baseline survey until death, loss-to follow-up or end of study, whichever happened first.

### Definition of ACEs

Information on early life ACEs before age 17 was collected through face-to-face interviews conducted during the 2014 life history survey. In alignment with prior research (Lin *et al.*, 2022), 10 ACE indicators were assessed, categorized into 2 domains: threat-related adversities (physical abuse, household substance abuse, domestic violence, neighbourhood violence and bullying) and deprivation-related adversities (emotional neglect, household mental illness, having an incarcerated household member, parental separation/divorce and parental death). The detailed definitions of individual ACE indicators are listed in the Supplementary Table 1.

Each indicator was binary-coded (0 = absent; 1 = present). Cumulative scores for threat-related and deprivation-related ACEs were calculated by summing the respective indicators within each domain. Participants were stratified into three groups per ACE dimension based on cumulative scores: 0, 1 or ≥2 threat-related ACEs, and analogously, 0, 1 or ≥2 deprivation-related ACEs.

### Definition of depressive and lonely

We defined depressive symptoms using the 10-item Center for Epidemiologic Studies Depression Scale (CES-D) at the baseline. The CES-D included 10 items measuring depressive symptoms: (1) bothered by little things; (2) had trouble concentrating; (3) felt depressed; (4) everything was an effort; (5) felt hopeful; (6) felt fearful; (7) sleep was restless; (8) felt happy; (9) felt lonely and (10) could not get going, with each item scoring from 0 (rarely or none of the time [<1 day] in the past week) to 3 (most or all of the time [5–7 days] in the past week). Depressive symptoms were defined as the total CES-D score ≥12 out of 30 (Gan *et al.*, 2025). We also used the item of feeling lonely to assess midlife loneliness, aligning with recent study (Wang *et al.*, 2024).

### Covariates

Referenced from previous investigations, several covariates were selected for adjustment (Arshadipour *et al.*, 2022; Chen *et al.*, 2024). These included sociodemographic characteristics, lifestyle behaviours and major chronic diseases. Sociodemographic characteristics included age (years), sex (male or female) and cohabitation status (living alone or not). Lifestyle behaviours included engaging in moderate-to-vigorous physical activities (no less than once weekly or not), regular alcohol consumption (at least once per week or not) and smoking status (current smoking or not). Major chronic conditions included physical disability and self-reported diagnosis of chronic diseases by physicians (Li *et al.*, 2025). Physical disability was defined as difficulty in one or more basic daily activities: bathing, dressing, eating, getting in/out of bed and using the toilet. Chronic diseases included hypertension, diabetes, cancer, chronic lung disease, heart disease, stroke and kidney disease. Considering some of the covariates could also serve as the mediators or colliders on the pathway from ACEs to GI/liver outcomes, the overall adjustment of covariates is descriptive. All covariates were measured at the baseline survey (2011).

### Statistical analysis

For descriptive statistics, the mean (standard deviation [SD]) was used for continuous variables, and numbers and percentages for categorical variables. Group differences were analysed using ANOVA or chi-squared test.

Cox proportional hazards regression models estimated the hazard ratios (HRs) and 95% confidence intervals (CIs) for chronic liver and GI diseases associated with threat-related and deprivation-related ACE exposure categories. We confirmed proportional hazards assumptions using weighted Schoenfeld residuals (*P* > 0.05). Linear trends were tested by treating ACE counts as continuous and estimated corresponding HRs and 95% CIs (Li *et al.*, 2022b), and linear dose–response effects with risks of chronic liver and GI diseases were further examined using ACEs as a continuous variable (0–5). Cumulative incidence of chronic liver and GI diseases, stratified by threat-related and deprivation-related ACEs, was estimated using Kaplan–Meier curves. The attained age during follow-up was chosen as the time scale for Cox regression and Kaplan–Meier analyses, to comprehensively account for the comprehensive relationships between aging and disease risks. Moreover, we simultaneously controlled for entry age as another covariate in the Cox regression model, aligning with our previous work (Liu and Li, 2025).

We estimated population attributable fractions (PAFs) to quantify the chronic liver/GI disease burden attributable to the threat-related and deprivation-related ACEs. Binary exposures of threat-related and deprivation-related ACEs were defined as the number of ACEs ≥ 2, to reflect the proportions of incident disease burden attributable to excessive ACE exposures. These estimates represent the attributable fraction under the specific model assumptions, including the assumed causal relationship between exposure and outcome. Using covariate-adjusted Cox regression models for time-to-event outcome, we calculated PAF via R package ‘AF’ (Elisabeth Dahlqwist, 2019).

We conducted mediation analysis to evaluate the mediation pathway of midlife depressive symptoms and loneliness in observed associations. Aligning with prior CHARLS cohort methodology (Gan *et al.*, 2025), we conducted mediation analysis using the difference approach, which can accommodate various mediators and the time-to-event outcome (Liu and Li, 2024). The mediation proportion was calculated by comparing HRs and 95% CIs from Cox regression models with versus without controlling for the mediators.

We conducted several sensitivity analyses to evaluate robustness of primary findings. First, to examine whether threat-related and deprivation-related ACEs independently influenced study outcomes, we controlled for number of deprivation-related ACEs in Cox models analysing threat-related ACEs and vice versa. Second, we adjusted for midlife socioeconomic status by including four variables in Cox regressions: annual family income, education qualifications, medical insurance coverage and employment status, following a previous study (Li *et al.*, 2025). Third, to address potential selection bias from participant exclusion, we applied inverse probability weighing (IPW; Li *et al.*, 2022a). In the IPW analysis, inclusion was predicted using baseline covariates: age, sex, cohabitation status, physical activity, alcohol consumption, current smoking, physical disability, hypertension, diabetes, cancer, chronic lung disease, heart disease, stroke and kidney disease. Weights were calculated as the inverse of probability of being included in analysis. Results were not materially changed after the IPW.

Following prior studies (Li *et al.*, 2022a, 2025), we used binary logistic regression to estimate inclusion probabilities, with major baseline characteristics included as the explanatory variables. Study samples were then re-weighted using these inverse probabilities. We assessed balance between included/excluded groups using absolute standardized mean differences (Love plot). Fourth, beyond the difference approach for mediation, we conducted causal mediation analysis under the counterfactual framework to account for exposure-mediator interactions (Zhang *et al.*, 2025). Fifth, non-response analysis compared baseline characteristics of included/excluded participants to evaluate selection bias. Sixth, aligning with our previous study (Li *et al.*, 2022b), we accounted for longitudinal changes of covariates in the analysis, using data from the waves 1 and 2 of the CHARLS cohort. The following covariates were adjusted in a time-varying fashion, including cohabitation status, physical activity, alcohol consumption, current smoking, physical disability, hypertension, diabetes, cancer, chronic lung disease, heart disease, stroke and kidney disease. Seventh, given the potential biases due to exclusions of baseline GI and liver diseases, we repeated the primary analysis by further retaining the prevalent cases in the analytical sample. Then, we used a modified Poisson regression to evaluate associations between exposure of ACEs and risks of GI and liver diseases, with risk ratio calculated (Zou, 2004). Eighth, compared to the 2011 survey as the baseline, the exposure of ACEs was evaluated during the 2014 life history survey, resulting in reverse causation challenge. Therefore, we repeated the primary analysis by further excluding new-onset GI and liver diseases that were recorded within 3 years since the baseline. Ninth, to clarify the rationale for the mediation analysis, we further examined the exposure-mediator associations and the mediator-outcome associations, respectively. For analytical purposes, the original loneliness score (ranging from 0 to 3) was transformed to a binary variable, with participants felt lonely ≥1 day during the last week defined as loneliness, aligning with a previous study (Wang *et al.*, 2024).

Statistical analysis was conducted using SAS 9.4 (SAS Institute, Cary, NC) and R language 4.3.1 (R Foundation, Vienna, Austria), with a two-tailed alpha of 0.05 considered statistically significant.

## Results

### Baseline characteristics

Of the 13,733 participants included in incident chronic liver diseases analysis, 6,446 (47.0%) were men and 7,287 (53.0%) were women (Table 1). Of these, 6,145 (44.7%) had experienced one or more threat-related ACEs, and 2,105 (15.3%) had been exposed to at least two childhood threats. Compared with no exposures, individuals with experience of at least two threat-related ACEs were more likely to be men (1,125 of 2,105 [53.4%] vs 3,246 of 7,588 [42.8%]) and younger (mean [SD] age, 56.9 [9.8] vs 58.9 [9.9] years), live alone (216 of 2,105 [10.3%] vs 938 of 7,588 [12.4%]), engage in less physical exercise (606 of 2,105 [28.8%] vs 1,963 of 7,588 [25.9%]) and have higher rates of current smoking (669 of 2,105 [31.8%] vs 2,002 of 7,588 [26.4%]), chronic lung disease (247 of 2,105 [11.7%] vs 627 of 7,588 [8.3%]) and hypertension (480 of 2,105 [22.8%] vs 1,986 of 7,588 [26.2%]).

**Table 1.** Baseline characteristics of participants for analysis of incident chronic liver diseases by number of threat-related ACEs and deprivation-related ACEs^a^ Table 1 long description.

	No. of threat-related ACEs				No. of deprivation-related ACEs			
Characteristics	0 (*n* = 7,588)	1 (*n* = 4,040)	≥2 (*n* = 2,105)	*P* for difference^b^	0 (*n* = 6,766)	1 (*n* = 5,487)	≥2 (*n* = 1,480)	*P* for difference^b^
Age, mean (SD), y	58.9 (9.9)	57.8 (9.6)	56.9 (9.8)	<0.001	57.7 (10.0)	58.5 (9.6)	59.9 (9.4)	<0.001
Sex				<0.001				0.279
Men	3,246 (42.8)	2,075 (51.4)	1,125 (53.4)		3,222 (47.6)	2,535 (46.2)	689 (46.6)	
Women	4,342 (57.2)	1,965 (48.6)	980 (46.6)		3,544 (52.4)	2,952 (53.8)	791 (53.4)	
Education				0.143				<0.001
Less than high school	6,779 (89.3)	3,607 (89.3)	1,889 (89.7)		5,916 (87.4)	4,964 (90.5)	1,395 (94.3)	
High school or equivalent	687 (9.1)	390 (9.7)	187 (8.9)		737 (10.9)	455 (8.3)	72 (4.9)	
College and higher	122 (1.6)	43 (1.1)	29 (1.4)		113 (1.7)	68 (1.2)	13 (0.9)	
Living alone	938 (12.4)	478 (11.8)	216 (10.3)	0.031	742 (11.0)	701 (12.8)	189 (12.8)	0.005
Physical exercise	1,963 (25.9)	1,104 (27.3)	606 (28.8)	0.017	1,795 (26.5)	1,478 (26.9)	400 (27.0)	0.851
Alcohol consumption	1,061 (14.0)	666 (16.5)	371 (17.6)	<0.001	1,023 (15.1)	837 (15.3)	238 (16.1)	0.647
Current smoking	2,002 (26.4)	1,223 (30.3)	669 (31.8)	<0.001	1,914 (28.3)	1,536 (28.0)	444 (30.0)	0.310
Physical disability	1,059 (14.0)	566 (14.0)	333 (15.8)	0.083	888 (13.1)	811 (14.8)	259 (17.5)	<0.001
Hypertension	1,986 (26.2)	980 (24.3)	480 (22.8)	0.002	1,689 (25.0)	1,383 (25.2)	374 (25.3)	0.941
Diabetes	432 (5.7)	226 (5.6)	131 (6.2)	0.578	390 (5.8)	316 (5.8)	83 (5.6)	0.972
Cancer	66 (0.9)	30 (0.7)	13 (0.6)	0.467	65 (1.0)	32 (0.6)	12 (0.8)	0.064
Chronic lung disease	627 (8.3)	386 (9.6)	247 (11.7)	<0.001	546 (8.1)	530 (9.7)	184 (12.4)	<0.001
Heart disease	887 (11.7)	413 (10.2)	227 (10.8)	0.049	732 (10.8)	610 (11.1)	185 (12.5)	0.176
Stroke	199 (2.6)	87 (2.2)	54 (2.6)	0.289	176 (2.6)	128 (2.3)	36 (2.4)	0.632
Kidney disease	361 (4.8)	219 (5.4)	126 (6.0)	0.049	329 (4.9)	289 (5.3)	88 (5.9)	0.200

Abbreviations: SD, standard deviation; ACE, adverse childhood experience.

a Unless indicated otherwise, data are expressed as no. (%) of participants.

b Group differences were tested using analysis of variance or chi-square test.

In terms of childhood deprivation, 6,967 participants (50.7%) were exposed to at least one deprivation-related ACEs and 1,480 participants (10.8%) reported exposure to two or more. Compared to those with no deprivation (*n* = 6,766), participants with two or more deprivation-related ACEs tended to be older (mean [SD] age, 59.9 [9.4] vs 57.7 [10.0] years) and had lower educational attainment (1,359 of 1,480 [94.3%] vs 5,916 of 6,766 [87.4%]). Notably, they were more likely to live alone, had higher rates of physical disability and chronic lung disease.

For the 10,661 participants included in incident GI disease analysis, 5,218 (48.9%) were men and 5,443 (51.1%) were women with a mean (SD) age of 58.3 (9.9) years at baseline. About 4,634 GI disease participants (43.5%) had experienced one or more threat-related ACE and 1,535 (14.4%) had exposure to at least two childhood threats. Compared with those without any exposure, participants who experienced two or more threat-related ACEs were younger, had a higher proportion of men, were more likely to live alone, consume alcohol, currently smoke and have chronic lung disease.

In terms of childhood deprivation, compared with the group without any deprivation during childhood, those who experienced two or more deprivation-related ACEs were significantly older and had lower educational attainment, and they were more likely to live alone (Table 2).

**Table 2.** Baseline characteristics of participants for analysis of incident gastrointestinal diseases by number of threat-related ACEs and deprivation-related ACEs^a^ Table 2 long description.

	No. of threat-related ACEs				No. of deprivation-related ACEs			
Characteristics	0 (*n* = 6,027)	1 (*n* = 3,099)	≥2 (*n* = 1,535)	*P* for difference^b^	0 (*n* = 5,420)	1 (*n* = 4,150)	≥2 (*n* = 1,091)	*P* for difference^b^
Age, mean (SD), y	58.9 (9.9)	57.8 (9.7)	57.1 (9.9)	<0.001	57.7 (10.0)	58.5 (9.7)	60.5 (9.6)	<0.001
Sex				<0.001				0.761
Men	2,684 (44.5)	1,662 (53.6)	872 (56.8)		2,652 (48.9)	2,021 (48.7)	545 (50.0)	
Women	3,343 (55.5)	1,437 (46.4)	663 (43.2)		2,768 (51.1)	2,129 (51.3)	546 (50.0)	
Education				0.199				<0.001
Less than high school	5,334 (88.5)	2,740 (88.4)	1,361 (88.7)		4,694 (86.6)	3,717 (89.6)	1,024 (93.9)	
High school or equivalent	584 (9.7)	322 (10.4)	148 (9.6)		625 (11.5)	374 (9.0)	55 (5.0)	
College and higher	109 (1.8)	37 (1.2)	26 (1.7)		101 (1.9)	59 (1.4)	12 (1.1)	
Living alone	751 (12.5)	374 (12.1)	148 (9.6)	0.009	593 (10.9)	529 (12.7)	151 (13.8)	0.003
Physical exercise	1,522 (25.3)	826 (26.7)	431 (28.1)	0.054	1,387 (25.6)	1,107 (26.7)	285 (26.1)	0.488
Alcohol consumption	889 (14.8)	548 (17.7)	282 (18.4)	<0.001	864 (15.9)	669 (16.1)	186 (17.0)	0.662
Current smoking	1,618 (26.8)	959 (30.9)	505 (32.9)	<0.001	1,541 (28.4)	1,189 (28.7)	352 (32.3)	0.035
Physical disability	754 (12.5)	373 (12.0)	206 (13.4)	0.407	643 (11.9)	529 (12.7)	161 (14.8)	0.026
Hypertension	1,579 (26.2)	776 (25.0)	354 (23.1)	0.036	1,375 (25.4)	1,057 (25.5)	277 (25.4)	0.994
Diabetes	355 (5.9)	182 (5.9)	94 (6.1)	0.934	317 (5.8)	248 (6.0)	66 (6.0)	0.949
Cancer	46 (0.8)	21 (0.7)	8 (0.5)	0.586	50 (0.9)	16 (0.4)	9 (0.8)	0.007
Chronic lung disease	473 (7.8)	276 (8.9)	160 (10.4)	<0.001	418 (7.7)	371 (8.9)	120 (11.0)	0.001
Heart disease	615 (10.2)	288 (9.3)	145 (9.4)	0.049	535 (9.9)	388 (9.3)	125 (11.5)	0.114
Stroke	159 (2.6)	72 (2.3)	47 (3.1)	0.289	144 (2.7)	102 (2.5)	32 (2.9)	0.646
Kidney disease	248 (4.1)	148 (4.8)	65 (4.2)	0.049	225 (4.2)	178 (4.3)	58 (5.3)	0.223

Abbreviations: SD, standard deviation; ACE, adverse childhood experience.

a Unless indicated otherwise, data are expressed as no. (%) of participants.

b Group differences were tested using analysis of variance or chi-square test.

### Associations between ACEs with risks of liver and GI diseases

**Figure 2.**
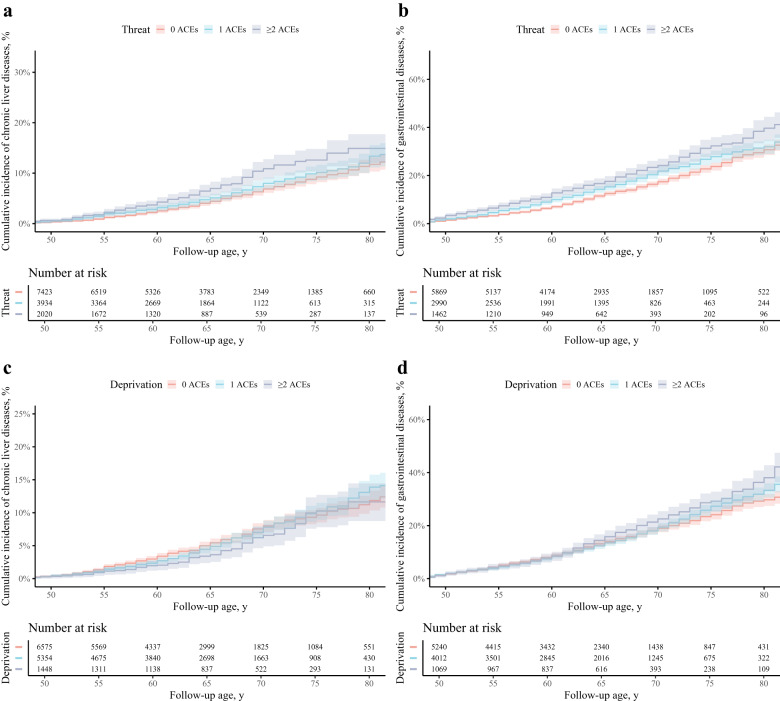
Crude cumulative incidence curves of chronic liver diseases and gastrointestinal diseases by number of threat-related and deprivation-related ACEs. (a) Cumulative incidence curve of chronic liver diseases by number of threat-related ACEs; (b) cumulative incidence curve of gastrointestinal diseases by number of threat-related ACEs; (c) cumulative incidence curve of chronic liver diseases by number of deprivation-related ACEs; (d) cumulative incidence curve of gastrointestinal diseases by number of deprivation-related ACEs. Figure 2 long description.

Figure 2 shows the cumulative incidence curves for chronic liver and GI diseases stratified by threat-related and deprivation-related ACEs. Threat-related ACEs show an increase in the incidence of both liver and GI diseases, and deprivation-related ACEs exhibit an even sharper increase in incidence specifically for GI outcomes.

ACE, adverse childhood experience. The number-at-risk table depicts the population at risk below each plot

The associations of threat-related and deprivation-related ACEs with incident chronic liver and GI diseases, respectively, were evaluated. Participants with experience of two or more threat-related ACEs were associated with chronic liver disease (HR, 1.26; 95% CI, 1.04–1.52; *P* = 0.016) and GI disease (HR, 1.36; 95% CI, 1.20–1.55; *P* < 0.001). A linear dose–response relationship was observed in liver disease (HR, 1.11; 95% CI, 1.03–1.20; *P* = 0.010; trend test *P* = 0.025) and GI disease (HR, 1.15; 95% CI, 1.09–1.21; *P* < 0.001; trend test *P* < 0.001). As for deprivation-related ACEs, no significant associations with liver disease risk across all exposure levels. In contrast, participants with experience of two or more deprivation-related ACEs showed a slightly higher effect size for GI disease (HR, 1.48; 95% CI, 1.28–1.70; *P* < 0.001), with consistent linear trends (HR, 1.18; 95% CI, 1.11–1.27; *P* < 0.001) (Table 3).

**Table 3.** Associations between threat-related ACEs and deprivation-related ACEs with incident chronic liver diseases and gastrointestinal diseases Table 3 long description.

	Incident chronic liver diseases			Incident gastrointestinal diseases		
Number of ACEs	No. of events/total	HR (95% CI)^a^	*P*-value	No. of events/total	HR (95% CI)^a^	*P-*value
**Threat-related ACEs**						
0 ACEs	404/7,588	1 [Reference]	[Reference]	935/6,027	1 [Reference]	[Reference]
1 ACEs	235/4,040	1.05 (0.89, 1.24)	0.546	541/3,099	1.14 (1.02, 1.27)	0.016
≥2 ACEs	152/2,105	1.26 (1.04, 1.52)	0.016	310/1,535	1.36 (1.20, 1.55)	<0.001
Linear trend test	791/13,733	1.11 (1.01, 1.22)	0.025	1,786/10,661	1.16 (1.09, 1.24)	<0.001
Per 1 ACE increment	791/13,733	1.11 (1.03, 1.20)	0.010	1,786/10,661	1.15 (1.09, 1.21)	<0.001
**Deprivation-related ACEs**						
0 ACEs	381/6,766	1 [Reference]	[Reference]	836/5,420	1 [Reference]	[Reference]
1 ACEs	325/5,487	1.04 (0.90, 1.21)	0.584	703/4,150	1.10 (1.00, 1.22)	0.055
≥2 ACEs	85/1,480	0.98 (0.77, 1.24)	0.872	247/1,091	1.48 (1.28, 1.70)	<0.001
Linear trend test	791/13,733	1.01 (0.91, 1.12)	0.888	1,786/10,661	1.18 (1.11, 1.27)	<0.001
Per 1 ACE increment	791/13,733	1.01 (0.91, 1.11)	0.920	1,786/10,661	1.18 (1.10, 1.25)	<0.001

Abbreviations: ACE, adverse childhood experience; HR, hazard ratio; CI, confidence interval.

a Cox proportional hazard regression was applied to estimate hazard ratios and 95% confidence intervals, controlling for age, sex, cohabitation status, physical activity, alcohol consumption, current smoking, physical disability, hypertension, diabetes, cancer, chronic lung disease, heart disease, stroke and kidney disease.

### Burden of chronic liver and GI diseases attributable to ACEs

**Figure 3.**
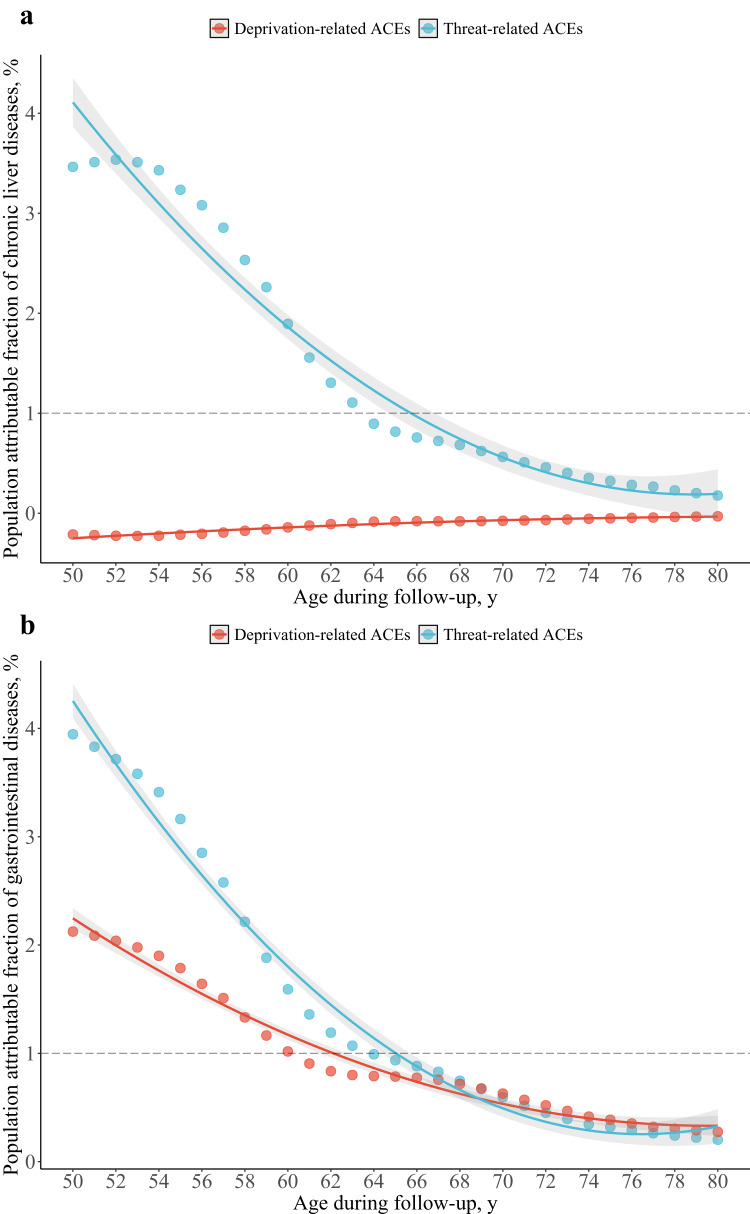
Population attributable fraction of incident chronic liver diseases and gastrointestinal disease. (a) Proportions of incident chronic liver diseases attributable to exposures of threat-related and deprivation-related ACEs. (b) Proportions of incident gastrointestinal diseases attributable to exposures of threat-related and deprivation-related ACEs. Figure 3 long description.

The estimation of age-dependent PAF revealed distinct trajectories for the two ACE clusters (Fig. 3). Age-dependent PAF for threat-related ACEs started at approximately 4.0% at age 50 for both outcomes, exhibiting a logarithmic decay towards zero by age 80. In contrast, deprivation-related ACEs showed negligible PAF for chronic liver disease (hovering near 0%) but contributed moderately to GI disease burden (starting at ∼2.1%), though consistently lower than threat-related ACEs in early adulthood.

ACE, adverse childhood experience. Binary ACE exposures were defined (threat-related ACEs ≥ 2 or not, deprivation-related ACEs ≥ 2 or not). Fitted Cox proportional hazard regression models with age as the time scale were used to estimate the attributable fraction for each exposure. Identical covariates in Tables 2 and 3 were adjusted for the analysis. Dots represent point estimates, while lines and shadows represent fitted smooth splines

### Mediation pathway of midlife

#### Midlife depression

**Figure 4.**
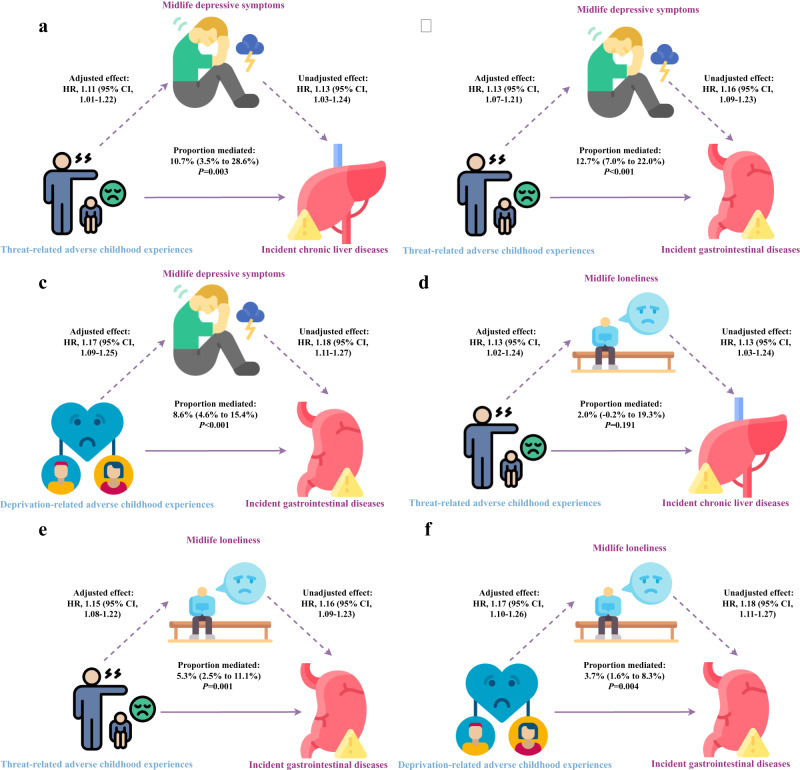
Mediation effect pathway of midlife depressive symptoms and loneliness in associations between ACEs with incident chronic liver diseases and gastrointestinal diseases. (a) Mediation effect of midlife depressive symptoms in associations between threat-related ACEs and incident chronic liver diseases; (b) mediation effect of midlife depressive symptoms in associations between threat-related ACEs and incident gastrointestinal diseases; (c) mediation effect of midlife depressive symptoms in associations between deprivation-related ACEs and incident gastrointestinal diseases; (d) mediation effect of midlife loneliness in associations between threat-related ACEs and incident chronic liver diseases; (e) mediation effect of midlife loneliness in associations between threat-related ACEs and incident gastrointestinal diseases; (f) mediation effect of midlife loneliness in associations between deprivation-related ACEs and incident gastrointestinal diseases. Figure 4 long description.

Mediation analysis identified midlife depressive symptoms as a partial mediator. For chronic liver disease, the association with threat-related ACEs was attenuated from a total effect HR of 1.13 (95% CI, 1.03–1.24) to a direct effect HR of 1.11 (95% CI, 1.01–1.22) after adjusting for depressive symptoms, corresponding to a mediation proportion of 10.7% (*P* = 0.003). Similarly, for GI disease, the HR decreased from 1.16 (total effect) to 1.13 (direct effect), and depressive symptoms accounted for 12.7% of the association (*P* < 0.001). For deprivation-related ACEs, depressive symptoms mediated 8.6% of the risk for GI disease (HR attenuation: 1.18–1.17; *P* < 0.001) (Fig. 4a–c).

Cox proportional hazard regression model was applied to estimate the hazard ratio and 95% CI of outcomes associated with ACEs. The unadjusted effect refers to the Cox model not controlling for the hypothesized mediator, while the adjusted effect refers to the Cox model controlling for midlife depressive symptoms or loneliness. Same covariates in Tables 2 and 3 were adjusted for all Cox models. Participants with missing measurements of depressive symptoms or loneliness score were excluded from the analysis

#### Midlife loneliness

The mediating role of loneliness was comparatively smaller. It statistically mediated the risk for GI disease conferred by threat-related ACEs (5.3% mediated; HR attenuation: 1.16–1.15; *P* = 0.001) and deprivation-related ACEs (3.7% mediated; HR attenuation: 1.18–1.17; *P* = 0.004). However, it is noteworthy that loneliness did not significantly mediate the relationship between threat-related ACEs and chronic liver disease (proportion mediated: 2.0%; *P* = 0.191), indicating pathway specificity (Fig. 4d–f).

### Supporting analyses

Several sensitivity analyses yield similar findings, supporting the robustness of our findings. These analyses are included in the Supplementary Tables 2–18 and Supplementary Figures 1**–**2. After accounting for time-varying covariates in our analysis, associations between threat-related ACEs and deprivation-related ACEs with incident GI diseases were not materially changed, while associations between ACEs and chronic liver diseases became less evident, as shown in Supplementary Tables 11 and 12. After retaining baseline cases of GI/liver diseases in our analysis, the overall findings were not materially changed, as shown in Supplementary Tables 13 and 14. After addressing potential reverse causation challenge in our analysis, the overall findings were not materially changed, as shown in Supplementary Tables 15 and 16. To support the interpretability of these mediated effects, we confirmed that both the exposure-mediator associations (ACEs and increased depressive symptoms) and mediator-outcome associations (depressive symptoms and incident GI/liver diseases) were statistically significant (Supplementary Tables 17 and 18), aligning with the insignificant mediation results reported in Fig. 4d.

## Discussion

Our study confirms that threat-related adversities increase the odds of having liver and GI diseases during the middle age, and a linear dose–response relationship was observed. In contrast, we did not find any association of deprivation-related ACEs with incident liver disease in midlife. Deprivation-related adversities are not universally linked to digestive disorders but are restricted to GI outcomes.

Notably, threat-related ACEs account for approximately 4% of the PAF for midlife chronic liver and GI diseases at age 50, with this contribution declining steadily with advancing age; deprivation-related ACEs contribute minimally to chronic liver disease PAF but account for ∼2% of GI disease PAF at age 50. This analysis identifies life stages where psychological screening may yield the greatest population benefit, supporting public health planning rather than definitive prediction. Importantly, PAF here reflects potential attributable burden, not definitive proof of causation; potential bias from unmeasured factors (e.g., genetics, socioeconomic background), even after adjusting for key confounders and using longitudinal data, further underscores this limitation and stronger causal evidence (e.g., from quasi-experimental studies) is therefore needed.

Prior research has shown associations between ACEs and increased risks for cardiovascular disease (Bengtsson *et al.*, 2023), metabolic disorders (Bengtsson *et al.*, 2021) and inflammatory burden (Rasmussen *et al.*, 2020). Although the health impacts of ACEs are well-documented, few studies have investigated the independent role of threat-related and deprivation-related ACEs on later-life GI and hepatic disorders in the general population, especially in terms of long-term consequences. To our knowledge, this study constitutes the first prospective analysis examining the correlation between ACEs and the incidence of GI and liver diseases in older adults. A prior cross-sectional study suggested a link between ACEs and IBS risk, but it did not distinguish between different dimensions of ACEs (Lee *et al.*, 2025). A Mendelian randomization study analysis also found that individuals exposed to childhood adversities had a 15% (95% CI, 1.12–1.19) higher risk of gastro-oesophageal diseases (Zhou *et al.*, 2025). Another study of the 1,172 liver transplant recipients, 24.1% endorsed a history of ACE, and those with a history of ACEs had a higher prevalence of hepatitis C virus (Fipps *et al.*, 2024). These prior studies, however, failed to disentangle the divergent effects of threat and deprivation subtypes, which may explain why the heterogeneous associations between deprivation-related ACEs and liver and GI outcomes (GI-positive, liver-null) have not been previously characterized. The present study further extends the findings of previous research by demonstrating the long-term association of threat-related ACEs with the rate of GI and liver diseases in later life and clarifies the GI-specific effect of deprivation-related ACEs that were obscured in aggregate ACE score analyses.

The dimensional model of early life adversity, particularly the distinction between threat and deprivation, provides a powerful theoretical framework for elucidating how different adverse experiences can lead to distinct neurobiological and health outcomes (Sheridan and McLaughlin, 2014; Mitchell *et al.*, 2025). This approach moves beyond cumulative risk scores to propose that the qualitative nature of adversity engages distinct psychobiological processes. It also highlights the limitations of total ACE scores, which obscure the unique influences of different childhood adversity types on mental health, cognitive development and long-term health outcomes.

Threat-related experiences are predominantly associated with alterations in neural circuits underlying fear learning and emotional processing, such as the amygdala and hippocampus. This type of adversity primarily dysregulates the hypothalamic–pituitary–adrenal (HPA) axis and fear-learning circuitry (McLaughlin *et al.*, 2014; Danese and Baldwin, 2017), resulting in persistent hypervigilant stress response. These responses present as heightened inflammatory reactivity and increased visceral sensitivity, which increase vulnerability to GI pathologies later in life (Sheridan and McLaughlin, 2014). Notably, this same HPA axis dysregulation and subsequent chronic inflammation disrupt hepatic lipid metabolism and promote steatosis, directly increasing chronic liver disease risk and offering a plausible explanation for our observation that a significant association between threat-related ACEs and GI and liver diseases.

In contrast, deprivation-related experiences are more strongly linked to structural and functional alterations in prefrontal cortical regions and associated impairments in executive functions (McLaughlin *et al.*, 2014, 2019; Sheridan and McLaughlin, 2014; McLaughlin, 2016; Danese and Baldwin, 2017). These neural changes impair executive functions, which in turn increase susceptibility to health-risk behaviours, including poor dietary choices and substance use (Hanson *et al.*, 2017). However, these behaviours may directly damage the intestinal mucosal barrier (e.g., high-fat, high-sugar diets disrupt gut microbiota balance), plausibly explaining why deprivation-related ACEs correlate with GI but not liver disease risk, as the indirect pathway lacks sufficient metabolic stress to induce hepatic pathology.

Our association between ACEs and liver and GI diseases was partially mediated by midlife depressive symptoms and loneliness. Although psychological role within this relationship has been described, our study is the first to investigate the extent of its mediating effect in liver and GI disease in national cohort study. Although ACEs elevate the odds of having liver and GI diseases, the risk may be further increased with depression and loneliness. The underlying molecular mechanism for this association remains indistinct. The scholars have proposed that threat-related ACEs may selectively impact neural circuits involved in emotional regulation, while deprivation-related ACEs appear to preferentially affect brain networks underlying language processing, executive control and cognitive engagement (Sheridan and McLaughlin, 2014). Early adversity or long-term stress can cause gut–brain axis imbalance, leading to impaired homeostasis of specific microbiota (Deng *et al.*, 2021). And an imbalance in the microbiome disrupts the intricate interplay between the brain, gut and liver (De Cól *et al.*, 2024). The liver serves as a key metabolic regulator, processing signals from the gut and fat tissue to manage nutrient metabolism. Gut-produced compounds (e.g., acetate) and adipose-derived fatty acids impact liver function (Steinberg *et al.*, 2025). Stress mediates GI symptoms by affecting gut function and microbiota composition via the HPA axis and reducing gut motility through the noradrenergic system, which might explain the significant effects of threat-related ACEs on liver and GI diseases.

Furthermore, chronic stress and negative emotions may potentially contribute to impaired synaptic plasticity and neural network dysfunction within the central nervous system, particularly in brain regions implicated in emotion regulation, autonomic nervous function and visceral sensory processing (e.g., the amygdala, hippocampus and prefrontal cortex) (McEwen, 2007). Persistent midlife loneliness, as a perceived state of social insecurity, could induce latent hypervigilance to social threats, thereby inducing chronic stress responses (Curtis *et al.*, 2025). While speculative, this heightened sensitivity to perceived stress and social threats in lonely individuals might lead to HPA-axis activation, hyperactive cortisol function and elevated inflammation, ultimately perpetuating immune dysregulation. This hypothetical pathway warrants further empirical validation in targeted mechanistic studies.

## Strengths and limitations

Our findings shed light on the long-term risk of incident in liver and GI diseases associated with ACEs based on a large-scale, high-quality, prospectively designed national longitudinal survey. Our study findings demonstrate that midlife depressive symptoms and loneliness partially mediate the association between ACEs and the risk of incident GI disease. From a clinical perspective, these findings might underscore the value of an integrated mental-physical care model in gastroenterological and hepatological care, where routine psychological screening in midlife could provide a more comprehensive risk profile for patients with a history of ACEs. Furthermore, we adopted a multidimensional framework to conceptualize ACEs and systematically investigated the differential effects of distinct adversity subtypes as well as dose–response associations between ACEs and GI and liver disease progression.

Nonetheless, this study also has several limitations. First, childhood ACEs were assessed in midlife using a self-reported questionnaire. This method may be susceptible to recall bias. Specifically, differential recall by mental state (e.g., mood-congruent memory bias) could occur, wherein individuals experiencing midlife depressive symptoms or loneliness might be more prone to reflect upon or over-report childhood adversities, which is especially problematic given mediation analysis. However, a previous study has clearly demonstrated that retrospective measurement of adverse experiences has good test-retest reliability, and compared with prospective measurement, it may provide some unique and complementary information. Second, participants were selected according to specific criteria, which may have introduced potential selection bias. However, we applied the IPW method to minimize residual confounding factors in the research findings as much as possible; nonetheless, it does not fully resolve selection effects. We acknowledge that residual confounding by cumulative life-course disadvantages cannot be completely ruled out in an observational design. Third, our outcome definitions represent broad categories based on self-reported diagnoses that explicitly excluded fatty liver, tumours or cancers. The lack of granularity to differentiate specific pathologies remains a limitation to be addressed by future studies with detailed clinical data. Fourth, mediator measurement validity is constrained by the use of a single item to define loneliness, as its overlap with the depression scale creates construct contamination. Fifth, our PAF calculations rely on the strong assumption of causality between ACEs and disease outcomes. As with any observational study, residual confounding and model misspecification could affect these estimates, which should therefore be interpreted as indicative of potential population health impact rather than definitive causal attributions. Sixth, factors like smoking and chronic diseases, measured at baseline, may lie on the causal pathway but were treated as confounders. This conservative approach may underestimate mediated effects, highlighting the need for future causal mediation analyses. Finally, although we applied different approaches for the mediation analysis and observed generally consistent findings, further cautions are needed to interpret such findings. As we cannot validate the underlying assumptions required for mediation analysis, such as no unmeasured confounding variables, correct model specification, etc., the risk of biased decomposition results persists. Further studies capable of appropriately handling the challenges are therefore needed to confirm our mediation findings.

## Conclusions

In this national prospective cohort study, we found that threat-related ACE exposures exhibit a direct association with an elevated risk of developing liver and GI diseases in adulthood. A modest proportion of this observed relationship is partially mediated through increased levels of depressive symptoms and feelings of loneliness experienced during midlife. We now explicitly emphasize the importance of routine psychological screening and the adoption of an integrated mental-physical care model. We have reframed midlife depression and loneliness as a critical ‘risk marker’ that warrants interdisciplinary attention, rather than a direct target for disease prevention.

## Supporting information

10.1017/S2045796026100729.sm001 Zhang et al. supplementary material Zhang et al. supplementary material

## Data Availability

All data generated or analysed during this study are included in this published article and its supplementary information files.

## Supplementary material

The supplementary material for this article can be found at https://doi.org/10.1017/S2045796026100729.

## Figure 1 Long description

The flowchart details the selection process of participants for a study. Initially, 17,708 participants attended wave 1. From these, 175 were excluded due to missing age and sex information, leaving 17,533 participants with complete demographic information. Next, 3,152 participants were excluded due to missing assessments of ACEs, resulting in 14,381 participants. From this group, 648 participants with chronic liver diseases at baseline or without follow-up were excluded, leading to 13,733 participants included in the incident chronic liver diseases analysis. Additionally, 3,720 participants with gastrointestinal diseases at baseline or without follow-up were excluded, resulting in 10,661 participants included in the incident gastrointestinal diseases analysis.

Navigate back to Figure 1.

## Figure 2 Long description

The image A showing a multi-line graph with the text “Threat” and legend entries “0 ACEs”, “1 ACEs” and “greater than or equal to 2 ACEs”. The horizontal axis label is “Follow-up age, y” with tick labels 50, 55, 60, 65, 70, 75, 80. The vertical axis label is “Cumulative incidence of chronic liver diseases, percent” with tick labels 0 percent, 10 percent, 20 percent, 30 percent. Three step-like lines rise from near 0 percent at age 50 toward higher values by age 80, with the line for “greater than or equal to 2 ACEs” above “1 ACEs” and “1 ACEs” above “0 ACEs” across the age range. Shaded bands surround each line. A table below the graph is labeled “Number at risk” with row label “Threat” and three rows of values aligned to 50, 55, 60, 65, 70, 75, 80: 7423, 6519, 5326, 3783, 2349, 1385, 660; 3934, 3364, 2669, 1864, 1122, 613, 315; 2020, 1672, 1320, 887, 539, 287, 137. The image B showing a multi-line graph with the text “Threat” and legend entries “0 ACEs”, “1 ACEs” and “greater than or equal to 2 ACEs”. The horizontal axis label is “Follow-up age, y” with tick labels 50, 55, 60, 65, 70, 75, 80. The vertical axis label is “Cumulative incidence of gastrointestinal diseases, percent” with tick labels 0 percent, 20 percent, 40 percent, 60 percent. Three step-like lines rise from near 0 percent at age 50 toward higher values by age 80, with the line for “greater than or equal to 2 ACEs” above “1 ACEs” and “1 ACEs” above “0 ACEs” across the age range. Shaded bands surround each line. A table below the graph is labeled “Number at risk” with row label “Threat” and three rows of values aligned to 50, 55, 60, 65, 70, 75, 80: 5869, 5137, 4174, 2935, 1857, 1095, 522; 2990, 2536, 1991, 1395, 826, 463, 244; 1462, 1210, 949, 642, 393, 202, 96. The image C showing a multi-line graph with the text “Deprivation” and legend entries “0 ACEs”, “1 ACEs” and “greater than or equal to 2 ACEs”. The horizontal axis label is “Follow-up age, y” with tick labels 50, 55, 60, 65, 70, 75, 80. The vertical axis label is “Cumulative incidence of chronic liver diseases, percent” with tick labels 0 percent, 5 percent, 10 percent, 15 percent, 20 percent, 25 percent. Three step-like lines rise from near 0 percent at age 50 toward higher values by age 80. The three lines are close together for much of the age range, with “greater than or equal to 2 ACEs” and “1 ACEs” near each other and “0 ACEs” also close. Shaded bands surround each line. A table below the graph is labeled “Number at risk” with row label “Deprivation” and three rows of values aligned to 50, 55, 60, 65, 70, 75, 80: 6575, 5569, 4337, 2999, 1825, 1084, 551; 5354, 4675, 3840, 2698, 1663, 908, 430; 1448, 1311, 1138, 837, 522, 293, 131. The image D showing a multi-line graph with the text “Deprivation” and legend entries “0 ACEs”, “1 ACEs” and “greater than or equal to 2 ACEs”. The horizontal axis label is “Follow-up age, y” with tick labels 50, 55, 60, 65, 70, 75, 80. The vertical axis label is “Cumulative incidence of gastrointestinal diseases, percent” with tick labels 0 percent, 20 percent, 40 percent, 60 percent. Three step-like lines rise from near 0 percent at age 50 toward higher values by age 80, with the line for “greater than or equal to 2 ACEs” above “1 ACEs” and “1 ACEs” above “0 ACEs” across the age range. Shaded bands surround each line. A table below the graph is labeled “Number at risk” with row label “Deprivation” and three rows of values aligned to 50, 55, 60, 65, 70, 75, 80: 5240, 4415, 3432, 2340, 1438, 847, 431; 4012, 3501, 2845, 2016, 1245, 675, 322; 1069, 967, 837, 616, 393, 238, 109.

Navigate back to Figure 2.

## Figure 3 Long description

The image A showing a line graph with markers and a legend. Legend text: Deprivation-related ACEs; Threat-related ACEs. Horizontal axis label: Age during follow-up, y. Horizontal axis range: 50 to 80. Vertical axis label: Population attributable fraction of chronic liver diseases, percent. Vertical axis range: 0 to 4. A horizontal dashed reference line is drawn at 1 on the vertical axis. Threat-related ACEs series coordinate pairs: (50, 3.5), (51, 3.5), (52, 3.5), (53, 3.5), (54, 3.5), (55, 3.3), (56, 3.1), (57, 2.9), (58, 2.5), (59, 2.3), (60, 1.9), (61, 1.6), (62, 1.3), (63, 1.1), (64, 0.9), (65, 0.8), (66, 0.8), (67, 0.7), (68, 0.6), (69, 0.6), (70, 0.5), (71, 0.5), (72, 0.4), (73, 0.4), (74, 0.3), (75, 0.3), (76, 0.3), (77, 0.2), (78, 0.2), (79, 0.2), (80, 0.2) Deprivation-related ACEs series coordinate pairs: (50, 0.2), (51, 0.2), (52, 0.2), (53, 0.2), (54, 0.2), (55, 0.2), (56, 0.2), (57, 0.2), (58, 0.2), (59, 0.2), (60, 0.2), (61, 0.2), (62, 0.2), (63, 0.2), (64, 0.2), (65, 0.2), (66, 0.2), (67, 0.2), (68, 0.2), (69, 0.2), (70, 0.2), (71, 0.2), (72, 0.2), (73, 0.2), (74, 0.2), (75, 0.2), (76, 0.2), (77, 0.2), (78, 0.2), (79, 0.2), (80, 0.2) The image B showing a line graph with markers and a legend. Legend text: Deprivation-related ACEs; Threat-related ACEs. Horizontal axis label: Age during follow-up, y. Horizontal axis range: 50 to 80. Vertical axis label: Population attributable fraction of gastrointestinal diseases, percent. Vertical axis range: 0 to 4. A horizontal dashed reference line is drawn at 1 on the vertical axis. Threat-related ACEs series coordinate pairs: (50, 4.0), (51, 3.8), (52, 3.7), (53, 3.5), (54, 3.3), (55, 3.1), (56, 2.8), (57, 2.5), (58, 2.2), (59, 1.9), (60, 1.6), (61, 1.4), (62, 1.2), (63, 1.1), (64, 1.0), (65, 0.9), (66, 0.8), (67, 0.8), (68, 0.7), (69, 0.6), (70, 0.5), (71, 0.5), (72, 0.4), (73, 0.4), (74, 0.3), (75, 0.3), (76, 0.3), (77, 0.3), (78, 0.3), (79, 0.2), (80, 0.2) Deprivation-related ACEs series coordinate pairs: (50, 2.1), (51, 2.0), (52, 2.0), (53, 1.9), (54, 1.8), (55, 1.7), (56, 1.6), (57, 1.5), (58, 1.3), (59, 1.2), (60, 1.0), (61, 0.9), (62, 0.9), (63, 0.8), (64, 0.8), (65, 0.8), (66, 0.7), (67, 0.7), (68, 0.7), (69, 0.6), (70, 0.6), (71, 0.5), (72, 0.5), (73, 0.4), (74, 0.4), (75, 0.4), (76, 0.3), (77, 0.3), (78, 0.3), (79, 0.3), (80, 0.3).

Navigate back to Figure 3.

## Figure 4 Long description

A 2 by 3 infographic showing six mediation pathways. Each pathway links adverse childhood experiences to a midlife mediator, then to an incident disease outcome. Each pathway includes text for adjusted effect, unadjusted effect and proportion mediated, alongside icons for an adult and child (ACEs), a seated figure (midlife depressive symptoms), a seated figure on a bench (midlife loneliness) and organ icons for liver or stomach. The top row shows threat-related adverse childhood experiences. Midlife depressive symptoms to incident chronic liver diseases: Adjusted effect: HR, 1.11 (95 percent CI, 1.01-1.22). Unadjusted effect: HR, 1.13 (95 percent CI, 1.03-1.24). Proportion mediated: 10.7 percent (3.5 percent to 28.6 percent). P=0.003. Midlife depressive symptoms to incident gastrointestinal diseases: Adjusted effect: HR, 1.13 (95 percent CI, 1.07-1.21). Unadjusted effect: HR, 1.16 (95 percent CI, 1.09-1.23). Proportion mediated: 12.7 percent (7.0 percent to 22.0 percent). P<0.001. Midlife loneliness to incident chronic liver diseases: Adjusted effect: HR, 1.13 (95 percent CI, 1.02-1.24). Unadjusted effect: HR, 1.13 (95 percent CI, 1.03-1.24). Proportion mediated: 2.0 percent (-0.2 percent to 19.3 percent). P=0.191. The bottom row includes one pathway for deprivation-related adverse childhood experiences and two additional pathways. Midlife depressive symptoms with deprivation-related adverse childhood experiences to incident gastrointestinal diseases: Adjusted effect: HR, 1.17 (95 percent CI, 1.09-1.25). Unadjusted effect: HR, 1.18 (95 percent CI, 1.11-1.27). Proportion mediated: 8.6 percent (4.6 percent to 15.4 percent). P<0.001. Midlife loneliness with threat-related adverse childhood experiences to incident gastrointestinal diseases: Adjusted effect: HR, 1.15 (95 percent CI, 1.08-1.22). Unadjusted effect: HR, 1.16 (95 percent CI, 1.09-1.23). Proportion mediated: 5.3 percent (2.5 percent to 11.1 percent). P=0.001. Midlife loneliness with deprivation-related adverse childhood experiences to incident gastrointestinal diseases: Adjusted effect: HR, 1.17 (95 percent CI, 1.10-1.26). Unadjusted effect: HR, 1.18 (95 percent CI, 1.11-1.27). Proportion mediated: 3.7 percent (1.6 percent to 8.3 percent). P=0.004.

Navigate back to Figure 4.

## Table 1 Long description

Baseline participant characteristics are compared across three groups by number of threat-related adverse childhood experiences and, separately, across three groups by number of deprivation-related adverse childhood experiences. With more threat-related ACEs, mean age decreases (about 58.9 to 56.9 years) and the proportion of men rises (about 42.8 to 53.4 percent). Threat-related ACEs also show higher alcohol consumption (about 14.0 to 17.6 percent), current smoking (about 26.4 to 31.8 percent), and chronic lung disease (about 8.3 to 11.7 percent), while hypertension is slightly lower (about 26.2 to 22.8 percent). Education levels are similar across threat-related ACE groups, and diabetes, cancer, and stroke vary little. For deprivation-related ACEs, mean age increases (about 57.7 to 59.9 years) and education shifts toward less than high school (about 87.4 to 94.3 percent), with fewer high school or college credentials. Deprivation-related ACEs are also associated with more physical disability (about 13.1 to 17.5 percent) and more chronic lung disease (about 8.1 to 12.4 percent), while sex, alcohol use, smoking, hypertension, diabetes, stroke, and kidney disease show minimal differences. Reported p-values indicate which group differences are statistically significant, but the table is descriptive and does not establish causation.

Navigate back to Table 1.

## Table 2 Long description

The table compares baseline participant characteristics across three groups by number of threat-related adverse childhood experiences (none, one, two or more) and separately across three groups by number of deprivation-related adverse childhood experiences. For threat-related ACEs, mean age decreases from 58.9 years (none) to 57.1 years (two or more), and the share of men rises from 44.5% to 56.8%. Smoking increases from 26.8% to 32.9% and alcohol consumption from 14.8% to 18.4% as threat-related ACE count increases; chronic lung disease also rises from 7.8% to 10.4%. Hypertension shows a small decline with more threat-related ACEs (26.2% to 23.1%), while diabetes and cancer are similar across groups. For deprivation-related ACEs, mean age increases from 57.7 years (none) to 60.5 years (two or more), and education shifts toward less than high school (86.6% to 93.9%) with fewer high school graduates (11.5% to 5.0%). Sex distribution is similar across deprivation-related ACE groups, but living alone increases (10.9% to 13.8%), smoking increases (28.4% to 32.3%), physical disability increases (11.9% to 14.8%), and chronic lung disease increases (7.7% to 11.0%). Several differences are statistically significant per the reported p values, but these are descriptive baseline comparisons and do not establish causation.

Navigate back to Table 2.

## Table 3 Long description

The table reports adjusted hazard ratios with 95% confidence intervals and p values for incident chronic liver disease and incident gastrointestinal disease by number of adverse childhood experiences, split into threat-related and deprivation-related groups. For threat-related ACEs, compared with zero ACEs, two or more ACEs had higher risk for chronic liver disease (HR 1.26, CI 1.04 to 1.52; p 0.016) and gastrointestinal disease (HR 1.36, CI 1.20 to 1.55; p less than 0.001). With one threat-related ACE, liver disease risk was not clearly different from the reference (HR 1.05; p 0.546), while gastrointestinal disease risk was higher (HR 1.14; p 0.016). Trend and per one ACE increment results for threat-related ACEs indicate increasing risk for both outcomes (liver HR about 1.11; gastrointestinal HR about 1.15 to 1.16, with p values 0.025 or smaller). For deprivation-related ACEs, chronic liver disease showed no clear association across categories (two or more ACEs HR 0.98; p 0.872; trend and per one ACE increment p values about 0.89 to 0.92). In contrast, deprivation-related ACEs were associated with higher gastrointestinal disease risk, especially for two or more ACEs (HR 1.48, CI 1.28 to 1.70; p less than 0.001), with significant trend and per one ACE increment results (HR about 1.18; p less than 0.001). Results are from Cox regression adjusted for demographic, lifestyle, and multiple health conditions, so they show associations rather than proof of causation.

Navigate back to Table 3.
