# Supplementary material for: Association of adverse childhood experiences and gastrointestinal and liver diseases among middle-aged and elderly adults in China
Source: Epidemiol Psychiatr Sci. 2026 Jul 20;35:e40. doi: 10.1017/S2045796026100729 (PMC13420166; doi:10.1017/S2045796026100729)
Supplement: Zhang et al. supplementary material [file S2045796026100729sup001.docx]

**Supplementary Tables and Figures**

**Supplementary Table 1**. Questionnaire Items of Each Threat-Related and Deprivation-Related ACE Indicator

[**Supplementary Table 2**. Associations between threat-related ACEs and deprivation-related ACEs with incident gastrointestinal diseases, mutually adjusted for each other.](#_Toc226056190)

[**Supplementary Table 3.** Associations between threat-related ACEs and deprivation-related ACEs with incident chronic liver diseases, further controlled for socioeconomic status indicators.](#_Toc226056191)

[S**upplementary Table 4**. Associations between threat-related ACEs and deprivation-related ACEs with incident gastrointestinal diseases, further controlled for socioeconomic status indicators.](#_Toc226056192)

[**Supplementary Table 5.** Associations between threat-related ACEs and deprivation-related ACEs with incident chronic liver diseases, based on inverse probability-weighted samples.](#_Toc226056193)

[**Supplementary Table 6.** Associations between threat-related ACEs and deprivation-related ACEs with incident gastrointestinal diseases, based on inverse probability-weighted samples.](#_Toc226056194)

[**Supplementary Table 7.** Causal mediation analysis of midlife depressive symptoms and loneliness in associations between ACEs with incident chronic liver diseases and gastrointestinal diseases.](#_Toc226056195)

[**Supplementary Table 8.** Non-response analysis comparing baseline characteristics between participants included and excluded from analysis of incident chronic liver diseases.](#_Toc226056196)

[**Supplementary Table 9.** Non-response analysis comparing baseline characteristics between participants included and excluded from analysis of incident gastrointestinal diseases.](#_Toc226056197)

[**Supplementary Table 11.** Associations between threat-related ACEs and deprivation-related ACEs with incident chronic liver diseases, controlling for time-varying covariates.](#_Toc226056198)

[**Supplementary Table 12.** Associations between threat-related ACEs and deprivation-related ACEs with incident gastrointestinal diseases, controlling for time-varying covariates.](#_Toc226056199)

[**Supplementary Table 13.** Associations between threat-related ACEs and deprivation-related ACEs with incident chronic liver diseases, retaining cases at the baseline.](#_Toc226056200)

[**Supplementary Table 14.** Associations between threat-related ACEs and deprivation-related ACEs with incident gastrointestinal diseases, retaining cases at the baseline.](#_Toc226056201)

[**Supplementary Table 15.** Associations between threat-related ACEs and deprivation-related ACEs with incident chronic liver diseases, excluding cases within 3 years since the baseline (year 2011) to address reverse causation.](#_Toc226056202)

[**Supplementary Table 16.** Associations between threat-related ACEs and deprivation-related ACEs with incident gastrointestinal diseases, excluding cases within 3 years since the baseline (year 2011) to address reverse causation.](#_Toc226056203)

[**Supplementary Table 17.** Exposure-mediator and mediator-outcome associations analysis regarding the incident chronic liver diseases outcome.](#_Toc226056204)

[**Supplementary Table 18.** Exposure-mediator and mediator-outcome associations analysis regarding the incident gastrointestinal diseases outcome.](#_Toc226056205)

**Supplementary Figure 1.** Love plot assessing the difference in baseline characteristics between participants included in and excluded from the analysis of incident chronic liver diseases, before and after inverse probability weighting.

**Supplementary Figure 2.** Love plot assessing the difference in baseline characteristics between participants included in and excluded from the analysis of incident gastrointestinal diseases, before and after inverse probability weighting.

**Supplementary Table 1.** Questionnaire Items of Each Threat-Related and Deprivation-Related ACE Indicator

| ACE indicators | Questionnaire Items and definitions |
| --- | --- |
| **Threat-related ACEs** | |
| Physical abuse | When you were growing up, did your female/male guardian ever hit you? (often^a^, sometimes^a^, rarely, or never) |
| Household substance abuse | During the years you were growing up, did your female/male guardian ever have alcoholism or drug? (yes^a^ or no) |
| Domestic violence | Have your father/mother ever beat up your mother/father? (often^a^, sometimes^a^, not very often, or never) |
| Unsafe neighborhood | Was it safe being out alone at night in the neighborhood where you lived as a child? (very safe, somewhat safe, not very safe^a^, or not safe at all^a^) |
| Bullying | 1) When you were a child, how often were you picked on or bullied by kids in your neighborhood? (often^a^, sometimes^a^, not very often, or never) |
|  | 2) When you were a child, how often were you picked on or bullied by kids in your school? (often^a^, sometimes^a^, not very often, or never) |
| **Deprivation-related ACEs** | |
| Emotional neglect | 1) How much love and affection did your female guardian give you while you were growing up? (often, sometimes, rarely^a^, or never^a^) |
|  | 2) How much effort did your female guardian put into watching over you? (a lot, some, a little^a^, or not at all^a^) |
| Household mental illness | 1) Did your female/male guardian have abnormality of mind when you were young? (yes^a^ or no) |
|  | 2) During the years you were growing up, had your female/male guardian often showed continued signs of sadness or depression? (during all^a^,most^a^, some, or only a little of the childhood) |
| Incarcerated  household member | During the years you were growing up, have your female/male guardian ever been arrested or sent to prison? (yes^a^ or no) |
| Parental separation or divorce | Were your biological parents divorced (including long separation due to emotional problems) before you were 17 years? (yes^a^ or no) |
| Parental deathb | Either of the parents was dead before participant was 17 years. (yes^a^ or no) |
| ^a^Answers indicate thresholds for ACEs.  ^b^Calculated based on dates of birth and their parental death. | |

**Supplementary Table 2.** Associations between threat-related ACEs and deprivation-related ACEs with incident chronic liver diseases, mutually adjusted for each other.

| **Number of ACEs** | **Incident chronic liver diseases** | | |
| --- | --- | --- | --- |
|  | **No. of events/total** | **HR (95% CI) ^a^** | ***P*-value** |
| **Threat-related ACEs** |  |  |  |
| 0 ACEs | 404/7588 | 1 [Reference] | [Reference] |
| 1 ACEs | 235/4040 | 1.05 (0.90, 1.24) | 0.536 |
| ≥ 2 ACEs | 152/2105 | 1.26 (1.05, 1.53) | 0.016 |
| Linear trend test | 791/13 733 | 1.11 (1.01, 1.22) | 0.025 |
| Per 1 ACE increment | 791/13 733 | 1.11 (1.03, 1.20) | 0.009 |
| **Deprivation-related ACEs** |  |  |  |
| 0 ACEs | 381/6766 | 1 [Reference] | [Reference] |
| 1 ACEs | 325/5487 | 1.02 (0.88, 1.19) | 0.757 |
| ≥ 2 ACEs | 85/1480 | 0.94 (0.74, 1.19) | 0.610 |
| Linear trend test | 791/13 733 | 0.99 (0.89, 1.10) | 0.812 |
| Per 1 ACE increment | 791/13 733 | 0.99 (0.89, 1.09) | 0.774 |

Abbreviations: ACE, adverse childhood experience; HR, hazard ratio; CI: confidence interval.

^a^ Cox proportional hazard regression was applied to estimate hazard ratios and 95% confidence intervals, controlling for age, sex, cohabitation status, physical activity, alcohol consumption, current smoking, physical disability, hypertension, diabetes, cancer, chronic lung disease, heart disease, stroke, and kidney disease. The analysis of threat-related ACEs was also adjusted for the number of deprivation-related ACEs and the analysis of deprivation-related ACEs was adjusted for the number of threat-related ACEs.

**Supplementary Table 3.** Associations between threat-related ACEs and deprivation-related ACEs with incident gastrointestinal diseases, mutually adjusted for each other.

| **Number of ACEs** | **Incident gastrointestinal diseases** | | |
| --- | --- | --- | --- |
|  | **No. of events/total** | **HR (95% CI) ^a^** | ***P*-value** |
| **Threat-related ACEs** |  |  |  |
| 0 ACEs | 935/6027 | 1 [Reference] | [Reference] |
| 1 ACEs | 541/3099 | 1.12 (1.00, 1.24) | 0.045 |
| ≥ 2 ACEs | 310/1535 | 1.31 (1.15, 1.49) | <0.001 |
| Linear trend test | 1786/10 661 | 1.14 (1.07, 1.21) | <0.001 |
| Per 1 ACE increment | 1786/10 661 | 1.13 (1.07, 1.19) | <0.001 |
| **Deprivation-related ACEs** |  |  |  |
| 0 ACEs | 836/5420 | 1 [Reference] | [Reference] |
| 1 ACEs | 703/4150 | 1.08 (0.98, 1.19) | 0.141 |
| ≥ 2 ACEs | 247/1091 | 1.41 (1.22, 1.62) | <0.001 |
| Linear trend test | 1786/10 661 | 1.16 (1.08, 1.24) | <0.001 |
| Per 1 ACE increment | 1786/10 661 | 1.15 (1.08, 1.23) | <0.001 |

Abbreviations: ACE, adverse childhood experience; HR, hazard ratio; CI: confidence interval.

^a^ Cox proportional hazard regression was applied to estimate hazard ratios and 95% confidence intervals, controlling for age, sex, cohabitation status, physical activity, alcohol consumption, current smoking, physical disability, hypertension, diabetes, cancer, chronic lung disease, heart disease, stroke, and kidney disease. The analysis of threat-related ACEs was also adjusted for the number of deprivation-related ACEs and the analysis of deprivation-related ACEs was adjusted for the number of threat-related ACEs.

**Supplementary Table 4.** Associations between threat-related ACEs and deprivation-related ACEs with incident chronic liver diseases, further controlled for socioeconomic status indicators.

| **Number of ACEs** | **Incident chronic liver diseases** | | |
| --- | --- | --- | --- |
|  | **No. of events/total** | **HR (95% CI) ^a^** | ***P*-value** |
| **Threat-related ACEs** |  |  |  |
| 0 ACEs | 404/7588 | 1 [Reference] | [Reference] |
| 1 ACEs | 235/4040 | 1.05 (0.90, 1.24) | 0.520 |
| ≥ 2 ACEs | 152/2105 | 1.27 (1.05, 1.53) | 0.013 |
| Linear trend test | 791/13 733 | 1.12 (1.02, 1.22) | 0.020 |
| Per 1 ACE increment | 791/13 733 | 1.11 (1.03, 1.20) | 0.008 |
| **Deprivation-related ACEs** |  |  |  |
| 0 ACEs | 381/6766 | 1 [Reference] | [Reference] |
| 1 ACEs | 325/5487 | 1.05 (0.90, 1.22) | 0.536 |
| ≥ 2 ACEs | 85/1480 | 0.99 (0.78, 1.26) | 0.943 |
| Linear trend test | 791/13 733 | 1.01 (0.91, 1.12) | 0.808 |
| Per 1 ACE increment | 791/13 733 | 1.01 (0.92, 1.11) | 0.843 |

Abbreviations: ACE, adverse childhood experience; HR, hazard ratio; CI: confidence interval.

^a^ Cox proportional hazard regression was applied to estimate hazard ratios and 95% confidence intervals, controlling for age, sex, cohabitation status, physical activity, alcohol consumption, current smoking, physical disability, hypertension, diabetes, cancer, chronic lung disease, heart disease, stroke, and kidney disease. Four indicators of socioeconomic status were also adjusted, including family annual income level (below the median or not), education qualifications, medical insurance coverage, and employment status.

**Supplementary Table 5.** Associations between threat-related ACEs and deprivation-related ACEs with incident gastrointestinal diseases, further controlled for socioeconomic status indicators.

| **Number of ACEs** | **Incident gastrointestinal diseases** | | |
| --- | --- | --- | --- |
|  | **No. of events/total** | **HR (95% CI) ^a^** | ***P*-value** |
| **Threat-related ACEs** |  |  |  |
| 0 ACEs | 935/6027 | 1 [Reference] | [Reference] |
| 1 ACEs | 541/3099 | 1.14 (1.02, 1.26) | 0.018 |
| ≥ 2 ACEs | 310/1535 | 1.35 (1.19, 1.54) | <0.001 |
| Linear trend test | 1786/10 661 | 1.16 (1.09, 1.23) | <0.001 |
| Per 1 ACE increment | 1786/10 661 | 1.15 (1.09, 1.21) | <0.001 |
| **Deprivation-related ACEs** |  |  |  |
| 0 ACEs | 836/5420 | 1 [Reference] | [Reference] |
| 1 ACEs | 703/4150 | 1.10 (0.99, 1.22) | 0.066 |
| ≥ 2 ACEs | 247/1091 | 1.47 (1.27, 1.69) | <0.001 |
| Linear trend test | 1786/10 661 | 1.18 (1.10, 1.26) | <0.001 |
| Per 1 ACE increment | 1786/10 661 | 1.17 (1.10, 1.25) | <0.001 |

Abbreviations: ACE, adverse childhood experience; HR, hazard ratio; CI: confidence interval.

^a^ Cox proportional hazard regression was applied to estimate hazard ratios and 95% confidence intervals, controlling for age, sex, cohabitation status, physical activity, alcohol consumption, current smoking, physical disability, hypertension, diabetes, cancer, chronic lung disease, heart disease, stroke, and kidney disease. Four indicators of socioeconomic status were also adjusted, including family annual income level (below the median or not), education qualifications, medical insurance coverage, and employment status.

**Supplementary Table 6.** Associations between threat-related ACEs and deprivation-related ACEs with incident chronic liver diseases, based on inverse probability-weighted samples.

| **Number of ACEs** | **Incident chronic liver diseases** | |
| --- | --- | --- |
|  | **HR (95% CI) ^a^** | ***P*-value** |
| **Threat-related ACEs** |  |  |
| 0 ACEs | 1 [Reference] | [Reference] |
| 1 ACEs | 1.03 (0.92, 1.16) | 0.577 |
| ≥ 2 ACEs | 1.25 (1.09, 1.43) | <0.001 |
| Linear trend test | 1.10 (1.03, 1.18) | 0.004 |
| Per 1 ACE increment | 1.10 (1.04, 1.16) | <0.001 |
| **Deprivation-related ACEs** |  |  |
| 0 ACEs | 1 [Reference] | [Reference] |
| 1 ACEs | 1.07 (0.96, 1.19) | 0.237 |
| ≥ 2 ACEs | 1.01 (0.86, 1.20) | 0.866 |
| Linear trend test | 1.03 (0.95, 1.10) | 0.495 |
| Per 1 ACE increment | 1.02 (0.95, 1.09) | 0.571 |

Abbreviations: ACE, adverse childhood experience; HR, hazard ratio; CI: confidence interval.

^a^ Cox proportional hazard regression was applied to estimate hazard ratios and 95% confidence intervals, controlling for age, sex, cohabitation status, physical activity, alcohol consumption, current smoking, physical disability, hypertension, diabetes, cancer, chronic lung disease, heart disease, stroke, and kidney disease. The inverse probability weighing approach was conducted to re-weight the original analytical samples to address the potential selection bias.

**Supplementary Table 7.** Associations between threat-related ACEs and deprivation-related ACEs with incident gastrointestinal diseases, based on inverse probability-weighted samples.

| **Number of ACEs** | **Incident gastrointestinal diseases** | |
| --- | --- | --- |
|  | **HR (95% CI) ^a^** | ***P*-value** |
| **Threat-related ACEs** |  |  |
| 0 ACEs | 1 [Reference] | [Reference] |
| 1 ACEs | 1.15 (1.06, 1.24) | 0.001 |
| ≥ 2 ACEs | 1.36 (1.23, 1.50) | <0.001 |
| Linear trend test | 1.16 (1.11, 1.22) | <0.001 |
| Per 1 ACE increment | 1.15 (1.10, 1.19) | <0.001 |
| **Deprivation-related ACEs** |  |  |
| 0 ACEs | 1 [Reference] | [Reference] |
| 1 ACEs | 1.10 (1.01, 1.19) | 0.020 |
| ≥ 2 ACEs | 1.49 (1.33, 1.66) | <0.001 |
| Linear trend test | 1.18 (1.12, 1.25) | <0.001 |
| Per 1 ACE increment | 1.18 (1.12, 1.24) | <0.001 |

Abbreviations: ACE, adverse childhood experience; HR, hazard ratio; CI: confidence interval.

^a^ Cox proportional hazard regression was applied to estimate hazard ratios and 95% confidence intervals, controlling for age, sex, cohabitation status, physical activity, alcohol consumption, current smoking, physical disability, hypertension, diabetes, cancer, chronic lung disease, heart disease, stroke, and kidney disease. The inverse probability weighing approach was conducted to re-weight the original analytical samples to address the potential selection bias.

**Supplementary Table 8.** Causal mediation analysis of midlife depressive symptoms and loneliness in associations between ACEs with incident chronic liver diseases and gastrointestinal diseases.

| **Exposures** | **Mediators** | **Outcomes** | **Effects decomposition (95% CI) ^a^** | | | **Proportion mediated (95% CI), %** | ***P*-value** |
| --- | --- | --- | --- | --- | --- | --- | --- |
|  |  |  | **TE** | **NDE** | **NIE** |  |  |
| Threat-related ACEs | Midlife depressive symptoms | Chronic liver diseases | 1.14 (1.03, 1.26) | 1.13 (1.02, 1.24) | 1.01 (1.00, 1.02) | 11.55 (0.53, 22.57) | 0.039 |
|  |  | Gastrointestinal diseases | 1.18 (1.10, 1.27) | 1.16 (1.07, 1.24) | 1.02 (1.01, 1.03) | 13.99 (6.39, 21.59) | <0.001 |
|  | Midlife loneliness | Chronic liver diseases | 1.14 (1.03, 1.26) | 1.14 (1.03, 1.25) | 1.00 (1.00, 1.01) | 0.69 (-3.03, 4.40) | 0.717 |
|  |  | Gastrointestinal diseases | 1.18 (1.10, 1.27) | 1.18 (1.09, 1.26) | 1.00 (1.00, 1.01) | 3.09 (0.14, 6.04) | 0.040 |
| Deprivation-related ACEs | Midlife depressive symptoms | Gastrointestinal diseases | 1.22 (1.13, 1.32) | 1.20 (1.11, 1.30) | 1.02 (1.01, 1.03) | 10.32 (4.70, 15.93) | <0.001 |
|  | Midlife loneliness | Gastrointestinal diseases | 1.22 (1.13, 1.32) | 1.22 (1.12, 1.31) | 1.00 (1.00, 1.01) | 2.31 (0.05, 4.58) | 0.044 |

Abbreviations: ACE, adverse childhood experience; TE, total effect; NDE, natural direct effect; NIE, natural indirect effect; CI: confidence interval.

^a^ Causal mediation effect analysis under the counter-factual framework was conducted for effect decomposition, with all effect estimates expressed as odds ratios and 95% confident intervals. Adjusted covariates included age, sex, cohabitation status, physical activity, alcohol consumption, current smoking, physical disability, hypertension, diabetes, cancer, chronic lung disease, heart disease, stroke, and kidney disease.

**Supplementary Table 9.** Non-response analysis comparing baseline characteristics between participants included and excluded from analysis of incident chronic liver diseases.

| **Characteristics** | **Excluded**  **n=3975** | **Included**  **n=13 733** | ***P* for difference ^a^** |
| --- | --- | --- | --- |
| Age, mean (SD), y | 59.4 (11.3) | 58.3 (9.8) | <0.001 |
| Sex |  |  | <0.001 |
| Men | 2032 (51.1%) | 6446 (46.9%) |  |
| Women | 1941 (48.9%) | 7287 (53.1%) |  |
| Education |  |  | <0.001 |
| Less than high school | 3286 (82.7%) | 12275 (89.4%) |  |
| High school or equivalent | 529 (13.3%) | 1264 (9.2%) |  |
| College and higher | 160 (4.0%) | 194 (1.4%) |  |
| Annual family income |  |  | <0.001 |
| Below the median | 1828 (46.0%) | 7067 (51.5%) |  |
| Above the median | 2147 (54.0%) | 6666 (48.5%) |  |
| Medical insurance coverage |  |  | <0.001 |
| Uninsured | 485 (12.2%) | 990 (7.2%) |  |
| Insured | 3490 (87.8%) | 12743 (92.8%) |  |
| Employment status |  |  | <0.001 |
| Unemployed | 276 (6.9%) | 587 (4.3%) |  |
| Employed | 3699 (93.1%) | 13146 (95.7%) |  |
| Living alone | 628 (15.8%) | 1632 (11.9%) | <0.001 |
| Physical exercise | 755 (19.0%) | 3673 (26.7%) | <0.001 |
| Alcohol consumption | 472 (11.9%) | 2098 (15.3%) | <0.001 |
| Current smoking | 977 (24.6%) | 3894 (28.4%) | <0.001 |
| Physical disability | 711 (17.9%) | 1958 (14.3%) | <0.001 |
| Hypertension | 1089 (27.4%) | 3446 (25.1%) | 0.004 |
| Diabetes | 273 (6.9%) | 789 (5.7%) | 0.010 |
| Cancer | 58 (1.5%) | 109 (0.8%) | <0.001 |
| Chronic lung disease | 444 (11.2%) | 1260 (9.2%) | <0.001 |
| Heart disease | 603 (15.2%) | 1527 (11.1%) | <0.001 |
| Stroke | 146 (3.7%) | 340 (2.5%) | <0.001 |
| Kidney disease | 273 (6.9%) | 706 (5.1%) | <0.001 |

Abbreviations: SD, standard deviation.

^a^ Group differences were tested using t-test or chi-square test.

**Supplementary Table 10.** Non-response analysis comparing baseline characteristics between participants included and excluded from analysis of incident gastrointestinal diseases.

| **Characteristics** | **Excluded**  **n=7047** | **Included**  **n=10 661** | ***P* for difference ^a^** |
| --- | --- | --- | --- |
| Age, mean (SD), y | 58.7 (10.6) | 58.3 (9.9) | 0.009 |
| Sex |  |  | 0.001 |
| Men | 3260 (46.3%) | 5218 (48.9%) |  |
| Women | 3785 (53.7%) | 5443 (51.1%) |  |
| Education |  |  | <0.001 |
| Less than high school | 6126 (86.9%) | 9435 (88.5%) |  |
| High school or equivalent | 739 (10.5%) | 1054 (9.9%) |  |
| College and higher | 182 (2.6%) | 172 (1.6%) |  |
| Annual family income |  |  | 0.660 |
| Below the median | 3525 (50.0%) | 5370 (50.4%) |  |
| Above the median | 3522 (50.0%) | 5291 (49.6%) |  |
| Medical insurance coverage |  |  | <0.001 |
| Uninsured | 656 (9.3%) | 819 (7.7%) |  |
| Insured | 6391 (90.7%) | 9842 (92.3%) |  |
| Employment status |  |  | 0.015 |
| Unemployed | 378 (5.4%) | 485 (4.5%) |  |
| Employed | 6669 (94.6%) | 10176 (95.5%) |  |
| Living alone | 987 (14.0%) | 1273 (11.9%) | <0.001 |
| Physical exercise | 1649 (23.4%) | 2779 (26.1%) | <0.001 |
| Alcohol consumption | 851 (12.1%) | 1719 (16.1%) | <0.001 |
| Current smoking | 1789 (25.4%) | 3082 (28.9%) | <0.001 |
| Physical disability | 1336 (19.0%) | 1333 (12.5%) | <0.001 |
| Hypertension | 1826 (25.9%) | 2709 (25.4%) | 0.465 |
| Diabetes | 431 (6.1%) | 631 (5.9%) | 0.611 |
| Cancer | 92 (1.3%) | 75 (0.7%) | <0.001 |
| Chronic lung disease | 795 (11.3%) | 909 (8.5%) | <0.001 |
| Heart disease | 1082 (15.4%) | 1048 (9.8%) | <0.001 |
| Stroke | 208 (3.0%) | 278 (2.6%) | 0.185 |
| Kidney disease | 518 (7.4%) | 461 (4.3%) | <0.001 |

Abbreviations: SD, standard deviation.

^a^ Group differences were tested using t-test or chi-square test.

**Supplementary Table 11.** Associations between threat-related ACEs and deprivation-related ACEs with incident chronic liver diseases, controlling for time-varying covariates.

| **Number of ACEs** | **Incident chronic liver diseases** | | |
| --- | --- | --- | --- |
|  | **No. of events/total** | **HR (95% CI) ^a^** | ***P*-value** |
| **Threat-related ACEs** |  |  |  |
| 0 ACEs | 404/7588 | 1 [Reference] | [Reference] |
| 1 ACEs | 235/4040 | 1.03 (0.88, 1.21) | 0.703 |
| ≥ 2 ACEs | 152/2105 | 1.21 (1.00, 1.46) | 0.051 |
| Linear trend test | 791/13 733 | 1.09 (0.99, 1.19) | 0.074 |
| Per 1 ACE increment | 791/13 733 | 1.09 (1.00, 1.17) | 0.037 |
| **Deprivation-related ACEs** |  |  |  |
| 0 ACEs | 381/6766 | 1 [Reference] | [Reference] |
| 1 ACEs | 325/5487 | 1.04 (0.90, 1.21) | 0.593 |
| ≥ 2 ACEs | 85/1480 | 0.96 (0.76, 1.21) | 0.727 |
| Linear trend test | 791/13 733 | 1.00 (0.90, 1.11) | 0.993 |
| Per 1 ACE increment | 791/13 733 | 1.00 (0.90, 1.10) | 0.964 |

Abbreviations: ACE, adverse childhood experience; HR, hazard ratio; CI: confidence interval.

^a^ Cox proportional hazard regression was applied to estimate hazard ratios and 95% confidence intervals, controlling for age, sex. The following covariates were adjusted in a time-varying fashion (based on data during wave 1 and 2), including cohabitation status, physical activity, alcohol consumption, current smoking, physical disability, hypertension, diabetes, cancer, chronic lung disease, heart disease, stroke, and kidney disease. The analysis of threat-related ACEs was also adjusted for the number of deprivation-related ACEs and the analysis of deprivation-related ACEs was adjusted for the number of threat-related ACEs.

**Supplementary Table 12.** Associations between threat-related ACEs and deprivation-related ACEs with incident gastrointestinal diseases, controlling for time-varying covariates.

| **Number of ACEs** | **Incident gastrointestinal diseases** | | |
| --- | --- | --- | --- |
|  | **No. of events/total** | **HR (95% CI) ^a^** | ***P*-value** |
| **Threat-related ACEs** |  |  |  |
| 0 ACEs | 935/6027 | 1 [Reference] | [Reference] |
| 1 ACEs | 541/3099 | 1.13 (1.02, 1.26) | 0.022 |
| ≥ 2 ACEs | 310/1535 | 1.34 (1.18, 1.53) | <0.001 |
| Linear trend test | 1786/10 661 | 1.15 (1.08, 1.23) | <0.001 |
| Per 1 ACE increment | 1786/10 661 | 1.14 (1.08, 1.20) | <0.001 |
| **Deprivation-related ACEs** |  |  |  |
| 0 ACEs | 836/5420 | 1 [Reference] | [Reference] |
| 1 ACEs | 703/4150 | 1.11 (1.00, 1.22) | 0.048 |
| ≥ 2 ACEs | 247/1091 | 1.46 (1.27, 1.69) | <0.001 |
| Linear trend test | 1786/10 661 | 1.18 (1.10, 1.26) | <0.001 |
| Per 1 ACE increment | 1786/10 661 | 1.17 (1.10, 1.25) | <0.001 |

Abbreviations: ACE, adverse childhood experience; HR, hazard ratio; CI: confidence interval.

^a^ Cox proportional hazard regression was applied to estimate hazard ratios and 95% confidence intervals, controlling for age, sex. The following covariates were adjusted in a time-varying fashion (based on data during wave 1 and 2), including cohabitation status, physical activity, alcohol consumption, current smoking, physical disability, hypertension, diabetes, cancer, chronic lung disease, heart disease, stroke, and kidney disease. The analysis of threat-related ACEs was also adjusted for the number of deprivation-related ACEs and the analysis of deprivation-related ACEs was adjusted for the number of threat-related ACEs.

**Supplementary Table 13.** Associations between threat-related ACEs and deprivation-related ACEs with incident chronic liver diseases, retaining cases at the baseline.

| **Number of ACEs** | **Incident chronic liver diseases** | | |
| --- | --- | --- | --- |
|  | **No. of events/total** | **Risk Ratio (95% CI) ^a^** | ***P*-value** |
| **Threat-related ACEs** |  |  |  |
| 0 ACEs | 705/7928 | 1 [Reference] | [Reference] |
| 1 ACEs | 397/4219 | 1.03 (0.92, 1.16) | 0.613 |
| ≥ 2 ACEs | 271/2234 | 1.27 (1.11, 1.45) | 0.000 |
| Linear trend test | 1373/14 381 | 1.11 (1.04, 1.19) | 0.002 |
| Per 1 ACE increment | 1373/14 381 | 1.11 (1.05, 1.18) | <0.001 |
| **Deprivation-related ACEs** |  |  |  |
| 0 ACEs | 653/7068 | 1 [Reference] | [Reference] |
| 1 ACEs | 556/5747 | 1.04 (0.93, 1.15) | 0.500 |
| ≥ 2 ACEs | 164/1566 | 1.09 (0.93, 1.28) | 0.309 |
| Linear trend test | 1373/14 381 | 1.04 (0.97, 1.12) | 0.281 |
| Per 1 ACE increment | 1373/14 381 | 1.04 (0.97, 1.12) | 0.231 |

Abbreviations: ACE, adverse childhood experience; HR, hazard ratio; CI: confidence interval.

^a^ Modified Poisson regression was applied to estimate risk ratios and 95% confidence intervals, controlling for age, sex, cohabitation status, physical activity, alcohol consumption, current smoking, physical disability, hypertension, diabetes, cancer, chronic lung disease, heart disease, stroke, and kidney disease. The analysis of threat-related ACEs was also adjusted for the number of deprivation-related ACEs and the analysis of deprivation-related ACEs was adjusted for the number of threat-related ACEs.

**Supplementary Table 14.** Associations between threat-related ACEs and deprivation-related ACEs with incident gastrointestinal diseases, retaining cases at the baseline.

| **Number of ACEs** | **Incident gastrointestinal diseases** | | |
| --- | --- | --- | --- |
|  | **No. of events/total** | **Risk Ratio (95% CI) ^a^** | ***P*-value** |
| **Threat-related ACEs** |  |  |  |
| 0 ACEs | 2806/7928 | 1 [Reference] | [Reference] |
| 1 ACEs | 1647/4219 | 1.11 (1.06, 1.17) | <0.001 |
| ≥ 2 ACEs | 1001/2234 | 1.26 (1.19, 1.33) | <0.001 |
| Linear trend test | 5454/14 381 | 1.12 (1.09, 1.15) | <0.001 |
| Per 1 ACE increment | 5454/14 381 | 1.10 (1.08, 1.13) | <0.001 |
| **Deprivation-related ACEs** |  |  |  |
| 0 ACEs | 836/5420 | 1 [Reference] | [Reference] |
| 1 ACEs | 703/4150 | 1.13 (1.08, 1.18) | <0.001 |
| ≥ 2 ACEs | 247/1091 | 1.28 (1.20, 1.36) | <0.001 |
| Linear trend test | 5454/14 381 | 1.13 (1.10, 1.16) | <0.001 |
| Per 1 ACE increment | 5454/14 381 | 1.12 (1.09, 1.15) | <0.001 |

Abbreviations: ACE, adverse childhood experience; HR, hazard ratio; CI: confidence interval.

^a^ Modified Poisson regression was applied to estimate risk ratios and 95% confidence intervals, controlling for age, sex, cohabitation status, physical activity, alcohol consumption, current smoking, physical disability, hypertension, diabetes, cancer, chronic lung disease, heart disease, stroke, and kidney disease. The analysis of threat-related ACEs was also adjusted for the number of deprivation-related ACEs and the analysis of deprivation-related ACEs was adjusted for the number of threat-related ACEs.

**Supplementary Table 15.** Associations between threat-related ACEs and deprivation-related ACEs with incident chronic liver diseases, excluding cases within 3 years since the baseline (year 2011) to address reverse causation.

| **Number of ACEs** | **Incident chronic liver diseases** | | |
| --- | --- | --- | --- |
|  | **No. of events/total** | **HR (95% CI) ^a^** | ***P*-value** |
| **Threat-related ACEs** |  |  |  |
| 0 ACEs | 312/7496 | 1 [Reference] | [Reference] |
| 1 ACEs | 192/3997 | 1.10 (0.92, 1.32) | 0.299 |
| ≥ 2 ACEs | 131/2084 | 1.40 (1.14, 1.72) | 0.001 |
| Linear trend test | 635/13 577 | 1.17 (1.06, 1.30) | 0.002 |
| Per 1 ACE increment | 635/13 577 | 1.15 (1.06, 1.26) | <0.001 |
| **Deprivation-related ACEs** |  |  |  |
| 0 ACEs | 300/6685 | 1 [Reference] | [Reference] |
| 1 ACEs | 263/5425 | 1.07 (0.91, 1.26) | 0.432 |
| ≥ 2 ACEs | 72/1467 | 1.05 (0.81, 1.36) | 0.703 |
| Linear trend test | 635/13 577 | 1.04 (0.93, 1.17) | 0.512 |
| Per 1 ACE increment | 635/13 577 | 1.04 (0.93, 1.16) | 0.527 |

Abbreviations: ACE, adverse childhood experience; HR, hazard ratio; CI: confidence interval.

^a^ Cox proportional hazard regression was applied to estimate hazard ratios and 95% confidence intervals, controlling for age, sex, cohabitation status, physical activity, alcohol consumption, current smoking, physical disability, hypertension, diabetes, cancer, chronic lung disease, heart disease, stroke, and kidney disease. The analysis of threat-related ACEs was also adjusted for the number of deprivation-related ACEs and the analysis of deprivation-related ACEs was adjusted for the number of threat-related ACEs.

**Supplementary Table 16.** Associations between threat-related ACEs and deprivation-related ACEs with incident gastrointestinal diseases, excluding cases within 3 years since the baseline (year 2011) to address reverse causation.

| **Number of ACEs** | **Incident gastrointestinal diseases** | | |
| --- | --- | --- | --- |
|  | **No. of events/total** | **HR (95% CI) ^a^** | ***P*-value** |
| **Threat-related ACEs** |  |  |  |
| 0 ACEs | 646/5738 | 1 [Reference] | [Reference] |
| 1 ACEs | 396/2954 | 1.19 (1.05, 1.35) | 0.006 |
| ≥ 2 ACEs | 202/1427 | 1.29 (1.10, 1.51) | 0.002 |
| Linear trend test | 1244/10 119 | 1.15 (1.06, 1.23) | <0.001 |
| Per 1 ACE increment | 1244/10 119 | 1.13 (1.06, 1.20) | <0.001 |
| **Deprivation-related ACEs** |  |  |  |
| 0 ACEs | 583/5167 | 1 [Reference] | [Reference] |
| 1 ACEs | 476/3923 | 1.07 (0.95, 1.21) | 0.246 |
| ≥ 2 ACEs | 185/1029 | 1.60 (1.35, 1.89) | <0.001 |
| Linear trend test | 1244/10 119 | 1.21 (1.12, 1.31) | <0.001 |
| Per 1 ACE increment | 1244/10 119 | 1.20 (1.12, 1.30) | <0.001 |

Abbreviations: ACE, adverse childhood experience; HR, hazard ratio; CI: confidence interval.

^a^ Cox proportional hazard regression was applied to estimate hazard ratios and 95% confidence intervals, controlling for age, sex, cohabitation status, physical activity, alcohol consumption, current smoking, physical disability, hypertension, diabetes, cancer, chronic lung disease, heart disease, stroke, and kidney disease. The analysis of threat-related ACEs was also adjusted for the number of deprivation-related ACEs and the analysis of deprivation-related ACEs was adjusted for the number of threat-related ACEs.

**Supplementary Table 17.** Exposure-mediator and mediator-outcome associations analysis regarding the incident chronic liver diseases outcome.

| **Explanatory variables** | **Response variables** | **OR/HR (95% CI) ^a^** | ***P*-value** |
| --- | --- | --- | --- |
| Per 1 threat-related ACE increment | Midlife depressive symptoms | 1.26 (1.20, 1.32) | <0.001 |
| Per 1 threat-related ACE increment | Midlife loneliness | 1.13 (1.08, 1.18) | <0.001 |
| Per 1 deprivation-related ACE increment | Midlife depressive symptoms | 1.27 (1.20, 1.34) | <0.001 |
| Per 1 deprivation-related ACE increment | Midlife loneliness | 1.16 (1.09, 1.22) | <0.001 |
| Midlife depressive symptoms | Incident chronic liver diseases | 1.31 (1.11, 1.54) | <0.001 |
| Midlife loneliness | Incident chronic liver diseases | 1.07 (0.90, 1.25) | 0.450 |

Abbreviations: ACE, adverse childhood experience; OR, odds ratio; HR, hazard ratio; CI: confidence interval.

^a^ Cox proportional hazard regression was applied to estimate hazard ratios and 95% confidence intervals (with incident chronic liver diseases as response variable), while the binary logistic regression applied to estimate odds ratios and 95% confidence intervals (with midlife depressive symptoms and loneliness as response variables). Covariates included age, sex, cohabitation status, physical activity, alcohol consumption, current smoking, physical disability, hypertension, diabetes, cancer, chronic lung disease, heart disease, stroke, and kidney disease.

**Supplementary Table 18.** Exposure-mediator and mediator-outcome associations analysis regarding the incident gastrointestinal diseases outcome.

| **Explanatory variables** | **Response variables** | **OR/HR (95% CI) ^a^** | ***P*-value** |
| --- | --- | --- | --- |
| Per 1 threat-related ACE increment | Midlife depressive symptoms | 1.27 (1.20, 1.34) | <0.001 |
| Per 1 threat-related ACE increment | Midlife loneliness | 1.14 (1.08, 1.21) | <0.001 |
| Per 1 deprivation-related ACE increment | Midlife depressive symptoms | 1.25 (1.17, 1.34) | <0.001 |
| Per 1 deprivation-related ACE increment | Midlife loneliness | 1.14 (1.07, 1.22) | <0.001 |
| Midlife depressive symptoms | Incident gastrointestinal diseases | 1.48 (1.33, 1.65) | <0.001 |
| Midlife loneliness | Incident gastrointestinal diseases | 1.39 (1.25, 1.54) | 0.004 |

Abbreviations: ACE, adverse childhood experience; OR, odds ratio; HR, hazard ratio; CI: confidence interval.

^a^ Cox proportional hazard regression was applied to estimate hazard ratios and 95% confidence intervals (with gastrointestinal diseases as response variable), while the binary logistic regression applied to estimate odds ratios and 95% confidence intervals (with midlife depressive symptoms and loneliness as response variables). Covariates included age, sex, cohabitation status, physical activity, alcohol consumption, current smoking, physical disability, hypertension, diabetes, cancer, chronic lung disease, heart disease, stroke, and kidney disease


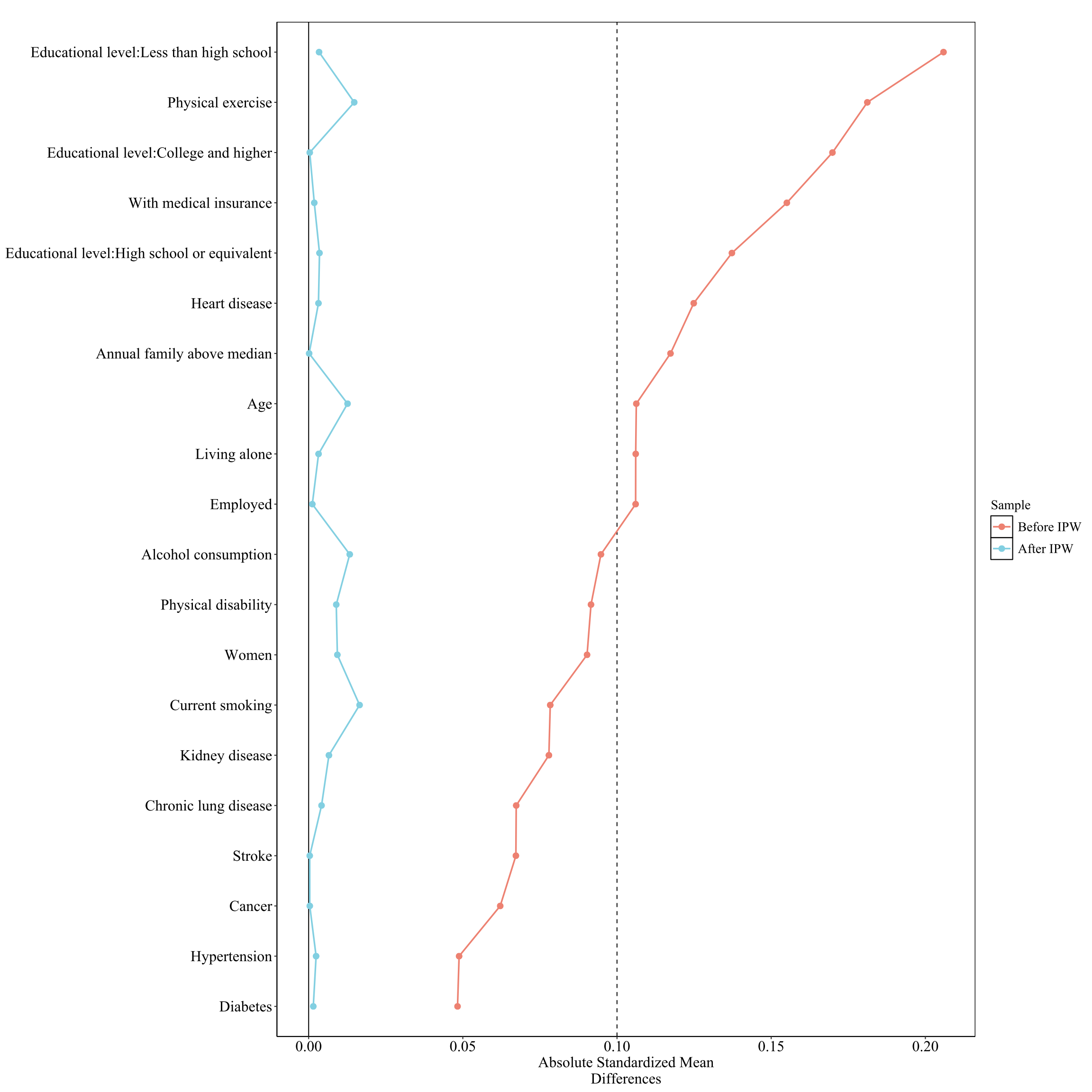


**Supplementary Figure 1.** Love plot assessing the difference in baseline characteristics between participants included in and excluded from the analysis of incident chronic liver diseases, before and after inverse probability weighting.

IPW, inverse probability weighting.

A binary logistic regression model including baseline variables was applied to estimate the probability of being included in the primary analysis for each participant, with the IPW weights applied to re-weight the sample. An absolute standardized mean difference of 0.1 was used to determine the significance of the imbalance.


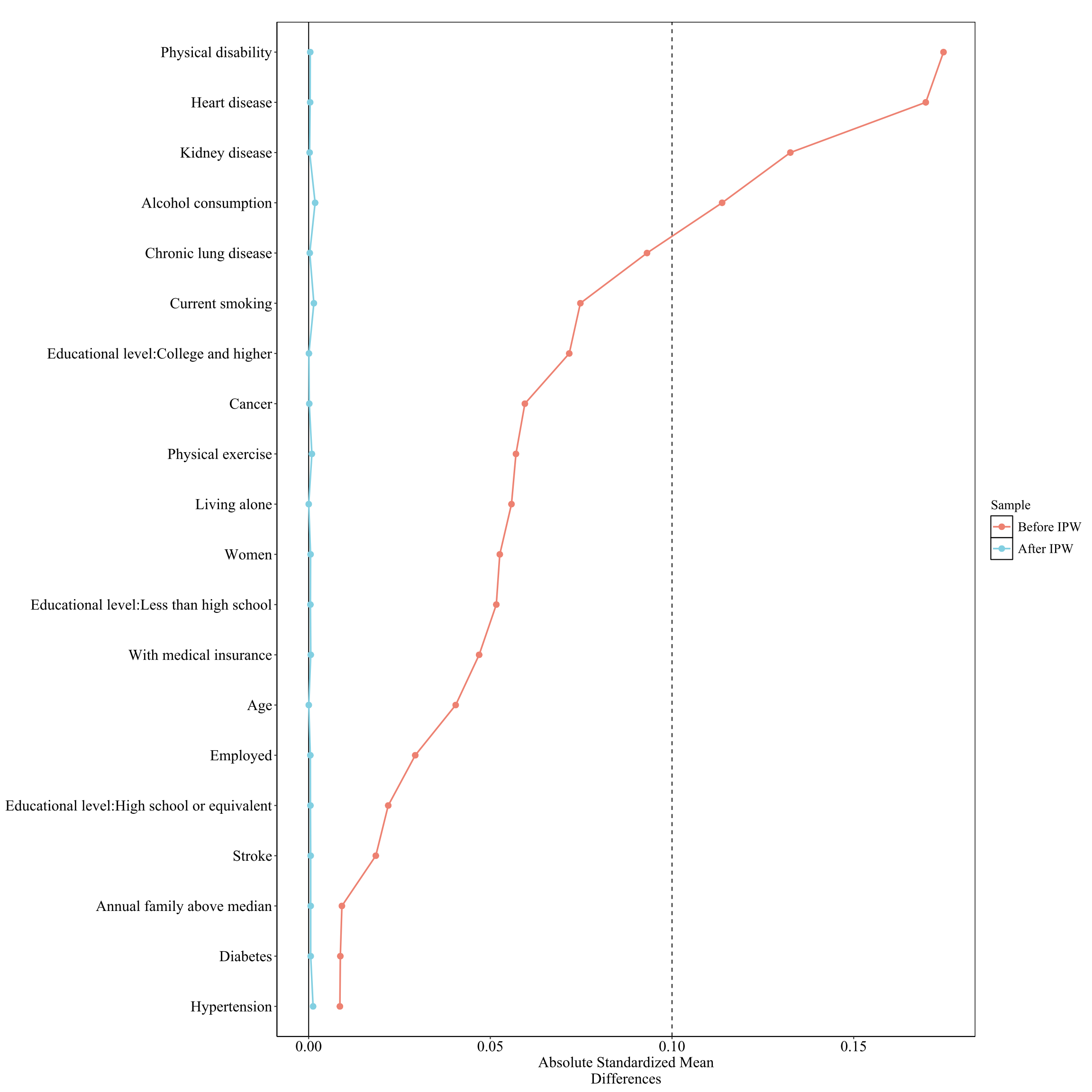


**Supplementary Figure 2.** Love plot assessing the difference in baseline characteristics between participants included in and excluded from the analysis of incident gastrointestinal diseases, before and after inverse probability weighting.

IPW, inverse probability weighting.

A binary logistic regression model including baseline variables was applied to estimate the probability of being included in the primary analysis for each participant, with the IPW weights applied to re-weight the sample. An absolute standardized mean difference of 0.1 was used to determine the significance of the imbalance.
